# Developing a codon optimization method for improved expression of recombinant proteins in actinobacteria

**DOI:** 10.1038/s41598-019-44500-z

**Published:** 2019-06-06

**Authors:** Yutaka Saito, Wataru Kitagawa, Toshitaka Kumagai, Naoyuki Tajima, Yoshiyuki Nishimiya, Koichi Tamano, Yoshiaki Yasutake, Tomohiro Tamura, Tomoshi Kameda

**Affiliations:** 1 Artificial Intelligence Research Center, National Institute of Advanced Industrial Science and Technology (AIST), 2-4-7 Aomi, Koto-ku, Tokyo, 135-0064 Japan; 2 Computational Bio Big-Data Open Innovation Laboratory (CBBD-OIL), National Institute of Advanced Industrial Science and Technology (AIST), 3-4-1 Okubo, Shinjuku-ku, Tokyo, 169-8555 Japan; 3 Bioproduction Research Institute, National Institute of Advanced Industrial Science and Technology (AIST), 2-17-2-1 Tsukisamu-Higashi, Toyohira-ku, Sapporo, 062-8517 Japan; 4 Graduate School of Agriculture, Hokkaido University, Kita 9-Nishi 9, Kita-ku, Sapporo, 060-8589 Japan; 5 Fermlab Inc., 913, 4-3-1 Shirakawa, Koto-ku, Tokyo, 135-0021 Japan

**Keywords:** Expression systems, Applied microbiology

## Abstract

Codon optimization by synonymous substitution is widely used for recombinant protein expression. Recent studies have investigated sequence features for codon optimization based on large-scale expression analyses. However, these studies have been limited to common host organisms such as *Escherichia coli*. Here, we develop a codon optimization method for *Rhodococcus erythropolis*, a gram-positive GC-rich actinobacterium attracting attention as an alternative host organism. We evaluate the recombinant protein expression of 204 genes in *R. erythropolis* with the same plasmid vector. The statistical analysis of these expression data reveals that the mRNA folding energy at 5’ regions as well as the codon frequency are important sequence features for codon optimization. Intriguingly, other sequence features such as the codon repetition rate show a different tendency from the previous study on *E. coli*. We optimize the coding sequences of 12 genes regarding these sequence features, and confirm that 9 of them (75%) achieve increased expression levels compared with wild-type sequences. Especially, for 5 genes whose expression levels for wild-type sequences are small or not detectable, all of them are improved by optimized sequences. These results demonstrate the effectiveness of our codon optimization method in *R. erythropolis*, and possibly in other actinobacteria.

## Introduction

Recombinant protein expression using bacterial and other host organisms is a fundamental technology for protein production^1^. A key step in recombinant protein expression is codon optimization where a coding sequence for a protein of interest is designed by synonymous substitution aiming to increase its expression level^2^.

Current approaches to codon optimization are based on sequence features considered to influence protein expression levels^3–6^. For example, a conventional approach is to substitute rare codons by frequent codons according to the genomic codon usage in a host organism. The basis of this approach is that endogenous genes whose coding sequences consist of frequent codons have high protein expression levels, and thus recombinant protein expression is also considered to be improved by increasing the codon frequency. Another approach is to introduce synonymous substitution computationally predicted to destabilize mRNA secondary structures. Since stable mRNA secondary structures may inhibit translation, this approach is considered to improve recombinant protein expression by enhancing translational efficiency. The association between these sequence features and protein expression levels has been indicated by omics analyses of endogenous genes (e.g.^7^). On the other hand, the direct evidence of their influences in recombinant protein expression has been shown using a relatively small number of genes (e.g.^8^).

Recently, large-scale analyses of recombinant protein expression have revealed sequence features for codon optimization in unprecedented detail^9,10^. In these studies, the recombinant protein expression of thousands of genes is evaluated in a systematic way using the same host organism and the same plasmid vector. Then, the influence of various sequence features on protein expression levels is investigated by statistical analyses. This strategy has provided new insights into conventionally-used sequence features such as the effect of the codon frequency depending on sequence positions (e.g. 5′ regions or others). In addition, a variety of new sequence features have been shown to be important including the use of specific di-codons and the repeated occurrence of codons in neighboring positions. However, such studies have been so far limited to common host organisms such as *Escherichia coli*, presenting a question whether these sequence features are also effective for codon optimization in less-studied host organisms.

*Rhodococcus erythropolis*, a gram-positive GC-rich actinobacterium, has been used as a host organism for recombinant protein expression and for the heterologous production of antimicrobial compounds^11–15^. *R. erythropolis* grows and produces recombinant proteins at a wide temperature range from 4 to 35 °C, and has different intracellular milieu compared to other host organisms such as *E. coli* (a gram-negative bacterium) and Bacillus and Lactococcus species (gram-positive bacteria with moderate GC contents). Due to these characteristics, *R. erythropolis* can produce recombinant proteins that are difficult to be expressed in *E. coli*. *R. erythropolis* has also been shown to produce the bacterial lipoglycoproteins from *Mycobacterium tuberculosis*, which cannot be expressed in *E. coli* for their post-translational modifications^16^. Based upon these performances, *R. erythropolis* is recognized as an alternative next-generation host microorganism.

Here, we develop a codon optimization method based on the statistical analysis of the recombinant protein expression data of 204 genes using *R. erythropolis* with the same plasmid vector. The statistical analysis reveals the mRNA folding energy at 5′ regions and the codon frequency as the most important sequence features for codon optimization. Interestingly, other sequence features including the codon repetition rate show a tendency different from *E. coli*, suggesting the species specificity of their influence on protein expression levels. We design the coding sequences of selected genes based on the optimization of these sequence features, and demonstrate that most of them become to show increased expression levels compared to wild-type sequences.

## Results

### Recombinant protein expression dataset in *R. erythropolis*

To develop a codon optimization method for *R. erythropolis*, we sought to investigate sequence features that influence protein expression levels. For this purpose, we evaluated the recombinant protein expression of 204 genes in *R. erythropolis* (Supplementary Data S1). These genes were selected from *Streptomyces coelicolor*, and were heterologously expressed in *R. erythropolis* using the pTip plasmid vector^12^. This expression system allowed us to evaluate the recombinant protein expression of various genes under the transcription from the same promoter, thereby focusing on their difference in translational efficiency. Based on the visual inspection of SDS-PAGE gels (Supplementary Fig. S1), protein expression levels were measured by integer scores: 1 (low or not detected), 2 (medium), and 3 (high). Note that such a discrete scoring scheme has also been employed in the previous study on *E. coli*^9^.

### Statistical analysis of sequence features influencing protein expression levels

**Figure 1:**
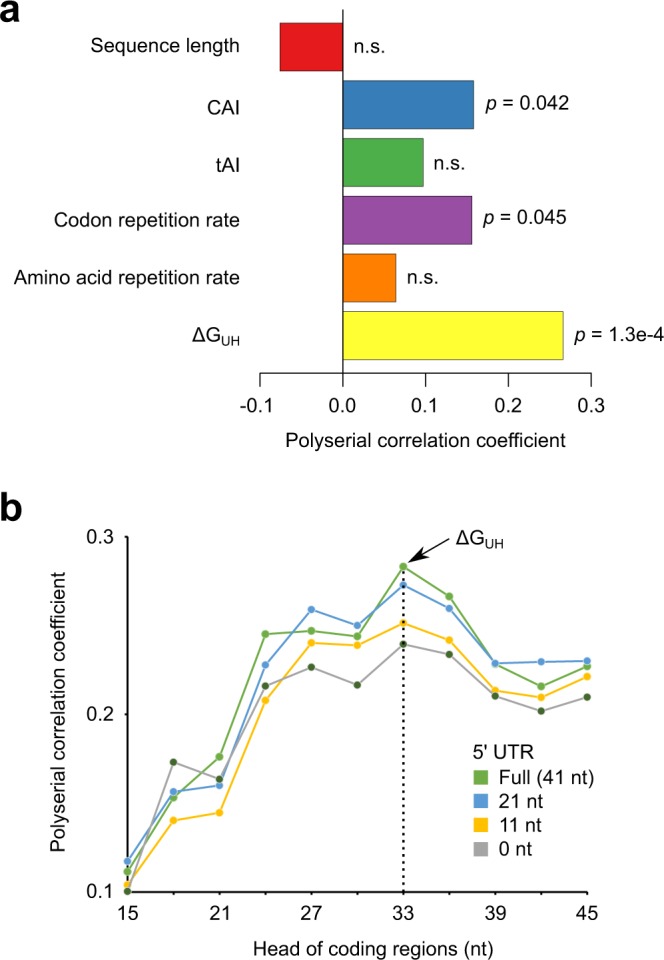
Influence of sequence features on protein expression levels. (**a**) For each type of sequence feature, a polyserial correlation coefficient between feature values and expression levels is shown with its p-value. The ΔG_UH_ gave the largest correlation coefficient, meaning that higher ΔG_UH_ (weaker mRNA secondary structures at 5′ regions) associate with higher expression levels. n.s.: *p* > 0.05. (**b**) The polyserial correlation coefficients for mRNA folding energies are shown using the different lengths of subsequences in 5′ UTR and the head coding region. The largest correlation coefficient was obtained when the full-length 5′ UTR and 33 head nucleotides were used, which is equivalent to ΔG_UH_ in (**a**).

We used our data to analyze the influence of sequence features on protein expression levels. We considered various sequence features including the measures of the codon frequency such as the codon adaptation index (CAI)^17^ and the tRNA adaptation index (tAI)^18^ and the measures of the repeated occurrence of codons such as the codon repetition rate^9^ and the amino acid repetition rate^9^. In addition, the stability of mRNA secondary structures at 5′ regions was measured by the folding energy (ΔG_UH_) predicted by EnsembleEnergy program in RNAStructure package^19^. For computing ΔG_UH_, 5′ regions were defined as the 5′ untranslated region (UTR) in the pTip plasmid vector plus 33 nucleotides at the head of coding sequences. Calculated feature values are summarized in Supplementary Data S1. For each type of sequence feature, a polyserial correlation coefficient^20^ was evaluated between feature values and protein expression levels (Fig. 1a). The positive correlation coefficients were detected for CAI, tAI, and ΔG_UH_, which is consistent with the previous report on *E. coli*^9^. Intriguingly, the results for the codon repetition rate and the amino acid repetition rate showed a tendency different from *E. coli*. While these sequence features have been reported to be negatively correlated with protein expression levels in *E. coli*, our results in *R. erythropolis* showed positive correlation coefficients, implicating the species-specific influence of these sequence features. Supplementary Fig. S2 summarizes the comparison between our results on *R. erythropolis* and the previous study on *E. coli*. CAI and ΔG_UH_ are important factors not only in *R. erythropolis* but also in *E. coli*. On the other hand, the contributions of the codon repetition rate and the amino acid repetition rate are larger in *E. coli* compared with *R. erythropolis*.

Among the sequence features considered, ΔG_UH_ showed the largest correlation coefficient, suggesting that higher mRNA folding energies (i.e. weaker mRNA secondary structures) at 5′ regions lead to increased protein expression levels. The influence of the mRNA folding energy was further investigated by changing the definition of 5′ regions (Fig. 1b). The correlation coefficient was the maximum for ΔG_UH_ with the full-length 5′ UTR plus 33 head nucleotides, whereas the use of extended or truncated 5′ regions did not achieve larger correlation coefficients. These results motivated us to develop a codon optimization method based on ΔG_UH_. In addition to ΔG_UH_, CAI showed the second-largest correlation coefficient, suggesting that the use of frequent codons is also effective for increasing protein expression levels.

### H-method: codon optimization based on mRNA folding energy

We first devised a codon optimization method based solely on ΔG_UH_, which we named “H-method”. For a given protein, H-method computationally generates coding sequences for all possible synonymous variants regarding to 33 head nucleotides. H-method then calculates ΔG_UH_ for each synonymous variant, and proposes the coding sequence with the highest ΔG_UH_.

We note that H-method introduces mutations only to 33 head nucleotides (i.e. 11 codons) with downstream nucleotides unmodified. Therefore, the number of generated synonymous variants can be kept relatively small, which allows us to calculate ΔG_UH_ for all possible synonymous variants regarding 33 head nucleotides. Such an exhaustive computation is not feasible when the entire coding sequence is mutated, since the number of possible synonymous variants increases exponentially with the sequence length. The merits of focusing on head nucleotides are not only computational costs but also experimental costs. If codon optimization modifies the entire coding sequence, we need to use full-length gene synthesis that requires a relatively high experimental cost. In contrast, the modification of head nucleotides can be performed by primer-based mutagenesis that is much cheaper than full-length gene synthesis (Methods). As will be shown later, this allows us to test the effectiveness of our method using a large number of sequences. Such a low experimental cost is important for facilitating a wide applicability of codon optimization.

**Figure 2:**
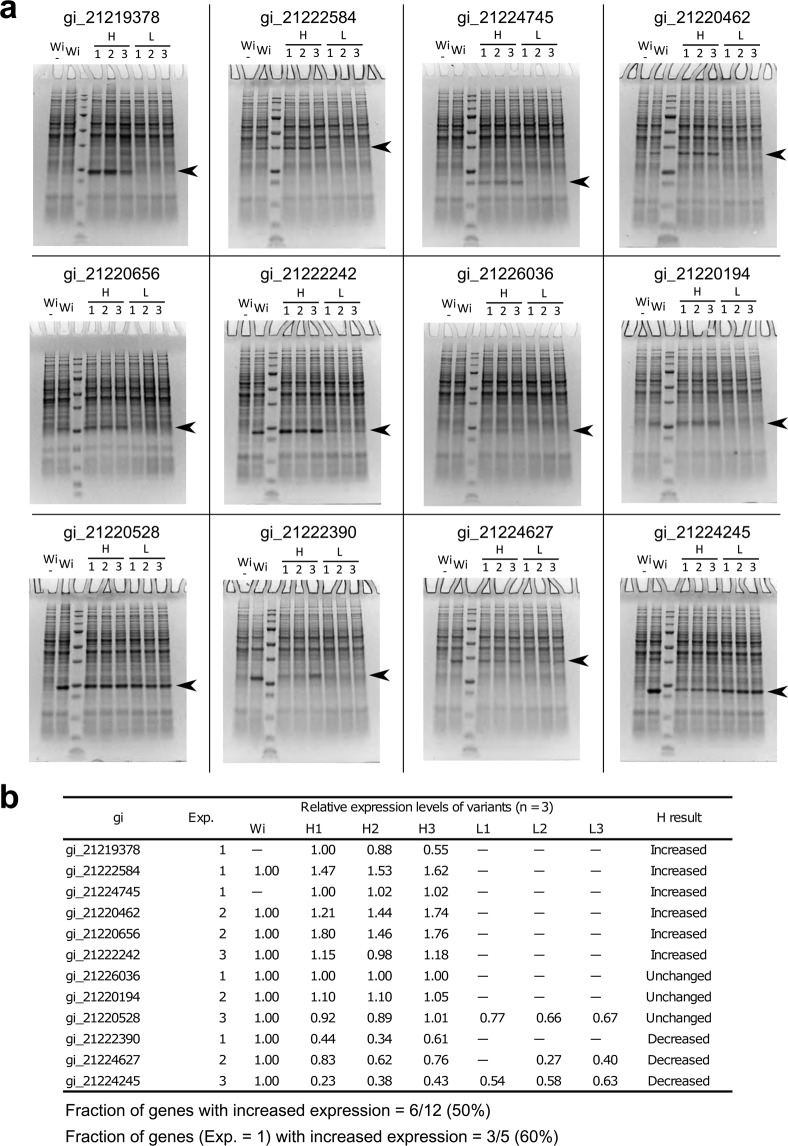
Codon optimization by H-method. (**a**) For each of 12 genes, the recombinant protein expression using different coding sequences is shown by SDS-PAGE. The expected positions of the recombinant proteins are indicated by black arrows. Wi: wild-type sequence. H1-3: optimized sequences with the first-to-third highest ΔG_UH_. L1-3: deoptimized sequences with the first-to-third lowest ΔG_UH_. W-: negative control where the wild-type sequence was used but the induction of expression was not performed. Note that although multiple gels are presented, the comparison of bands is performed only within each gel, but not between different gels. No gels are cropped and regrouped for comparison. For confirmation, raw image data are available as Supplementary Data S5. (**b**) Expression levels for the optimized sequences relative to the wild-type sequence. For gi_21219378 and gi_21224745, expression levels relative to H1 are shown since the expression for the wild-type sequences was not detected. Each expression level is the average of three biological replicates from individual recombinant clones. —: expression not detected. Also shown is the summary of the results for the H-series sequences. Increased: more than 1.1-fold increase. Decreased: less than 0.9-fold decrease. Unchanged: not changed by means of the threshold of the 1.1-fold or 0.9-fold change. Exp.: integer expression score for the wild-type sequence in our expression dataset (Supplementary Data S1).

To test the effectiveness of H-method, we design the coding sequences of genes using H-method, and compared protein expression levels between the optimized sequences and the wild-type sequences. We selected 12 genes so that their protein expression levels before codon optimization vary: 5 genes with the score of 1 (low or not detected), 4 genes with the score of 2 (medium), and 3 genes with the score of 3 (high). For each gene, we designed the 3 sequences with the first-to-third highest ΔG_UH_ using H-method (H1-3). For comparison, we also designed the 3 sequences with the first-to-third lowest ΔG_UH_ (i.e. deoptimized sequences; L1-3). These sequences were transformed into *R. erythropolis* using the pTip plasmid vector (Fig. 2a), and the protein expression levels were measured using three biological replicates (Fig. 2b). In summary, the optimized sequences showed increased expression levels compared with the wild-type sequences for 6 out of the 12 genes. However, for the remaining 6 genes, the improvement in protein expression was not observed. These results demonstrated the effectiveness of H-method, while indicating its limitation in the success rate. The effect of ΔG_UH_ in codon optimization was also supported by the deoptimized sequences whose protein expression levels were substantially decreased from the wild-type sequences for all of the 12 genes.

**Figure 3:**
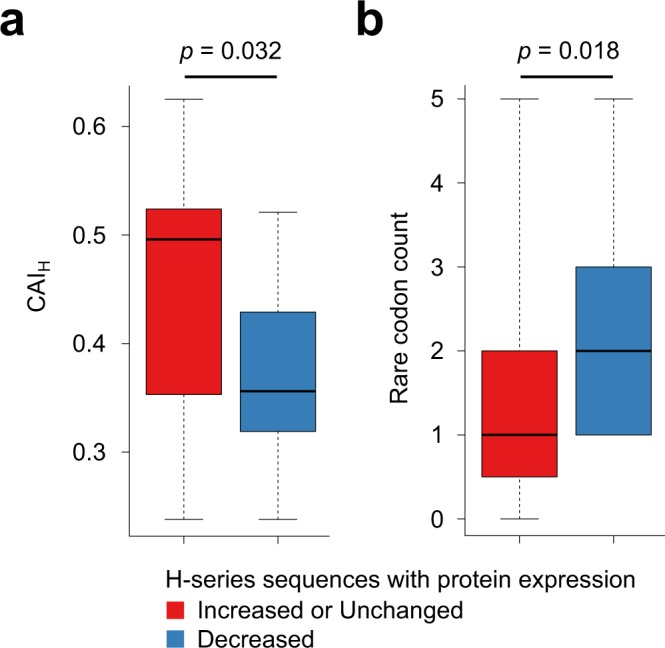
Failure of expression improvement by H-method is associated with decreased codon frequencies. For the H-series sequences, CAI_H_ (**a**) and the rare codon count (**b**) are compared between genes whose expression levels were decreased by H-method (indicated as “Decreased” in Fig. 2b) or not (indicated as “Increased” or “Unchanged” in Fig. 2b). P-values were calculated by Mann-Whitney *U* test.

For investigating the cause that H-method could not improve protein expression, we recall that CAI showed the second-largest correlation coefficient next to ΔG_UH_ (Fig. 1a). Therefore, we hypothesized that the mutations introduced by H-method might decrease CAI, thereby leading to reduced protein expression levels. To test this hypothesis, we calculated CAI for the coding sequences designed by H-method focusing only on 33 head nucleotides (CAI_H_). CAI_H_ was compared between the two groups of genes whose protein expression levels were decreased or not (Fig. 3a). We also measured the number of rare codons used in 33 head nucleotides (Fig. 3b; Supplementary Data S2) where rare codons were defined as TTA, ATA, and AGA whose normalized codon frequencies are less than 0.1 (Methods). Indeed, the coding sequences designed by H-method showed smaller CAI_H_ and more rare codons for the genes with decreased protein expression levels compared to the other genes. These results suggested that a more powerful codon optimization method can be developed by combining both ΔG_UH_ and CAI_H_.

### C-method: codon optimization combining mRNA folding energy and codon frequency

To improve the success rate of our codon optimization method, we next devised an approach combining both ΔG_UH_ and CAI_H_, which we named “C-method”. Similar to H-method, C-method proposes the coding sequence with the highest ΔG_UH_ from synonymous variants regarding 33 head nucleotides. However, generated synonymous variants are restricted so that they have CAI_H_ larger than a user-specified threshold and contain no rare codon. This approach enables us to design coding sequences with high ΔG_UH_ while controlling CAI_H_ (Supplementary Fig. S3).

**Figure 4:**
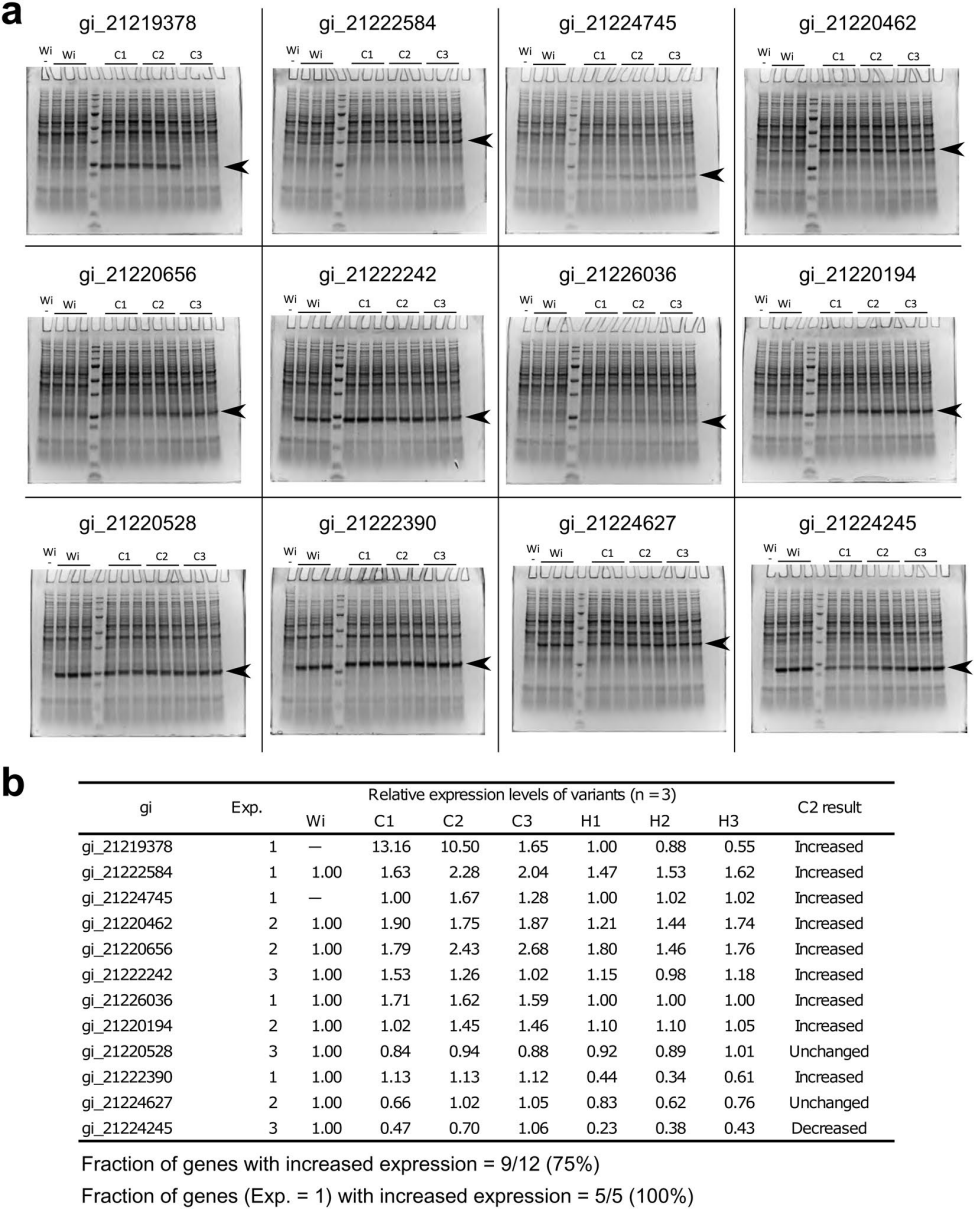
Codon optimization by C-method. (**a**) For each of 12 genes, the recombinant protein expression using different coding sequences is shown by SDS-PAGE. The expected positions of the recombinant proteins are indicated by black arrows. Wi: wild-type sequence. C1-3: optimized sequences with the highest ΔG_UH_ satisfying the CAI_H_ thresholds of 0.60 (C1), 0.75 (C2), and 0.90 (C3). H1-3: optimized sequences with the first-to-third highest ΔG_UH_ without the restriction of CAI_H_. W-: negative control where the wild-type sequence was used but the induction of expression was not performed. Note that although multiple gels are presented, the comparison of bands is performed only within each gel, but not between different gels. No gels are cropped and regrouped for comparison. For confirmation, raw image data are available as Supplementary Data S5. (**b**) Expression levels for the optimized sequences relative to the wild-type sequence. For gi_21219378 and gi_21224745, expression levels relative to H1 are shown since the expression for the wild-type sequences was not detected as indicated by “—”. Each expression level is the average of three biological replicates from individual recombinant clones. Increased: more than 1.1-fold increase. Decreased: less than 0.9-fold decrease. Unchanged: not changed by means of the threshold of the 1.1-fold or 0.9-fold change. Exp.: integer expression score for the wild-type sequence in our expression dataset (Supplementary Data S1).

We tested the effectiveness of C-method by an experiment similar to that for H-method (Fig. 4a). For each gene, the 3 sequences were designed by C-method using the different thresholds for CAI_H_: 0.60 (C1), 0.75 (C2), and 0.90 (C3). C-method achieved the success rate better than H-method (Fig. 4b). For the C2 sequences, protein expression levels were increased for 9 out of the 12 genes (75%). Strikingly, when focusing on the 5 genes whose protein expression levels for the wild-type sequences were low or not detectable, all of them were improved by optimized sequences. For the C1 and C3 sequences, 8 out of the 12 genes were improved in each case, showing the success rate slightly worse than the C2 sequences. However, the improvement was still observed for all of the 5 genes with poor wild-type expression levels. These results demonstrated the effectiveness of C-method for improved expression of recombinant proteins in *R. erythropolis*.

**Figure 5:**
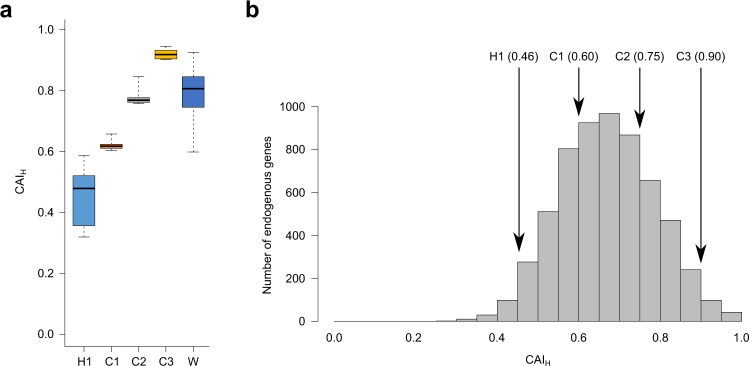
Selecting CAI_H_ thresholds for C-method. (**a**) CAI_H_ of the sequences designed by C-method are compared with the wild-type sequences. C1-3: sequences designed with the CAI_H_ thresholds of 0.60 (C1), 0.75 (C2), and 0.90 (C3). W: wild-type sequences. H1: sequences designed by H-method. (**b**) CAI_H_ thresholds are compared with the distribution of CAI_H_ computed from the all endogenous genes in *R. erythropolis*. For H1, the mean CAI_H_ of designed sequences (0.46) is shown.

Since C-method uses a CAI_H_ threshold as a parameter, we need the criteria for selecting the CAI_H_ threshold when applying C-method to other genes and/or host organisms. For this purpose, we conducted the following analyses. First, we compared CAI_H_ of the C1, C2, and C3 sequences with the corresponding wild-type sequences before codon optimization (Fig. 5a). We found that the C2 sequences had on average a similar level of CAI_H_ to the wild-type sequences while the C1 and C3 sequences were at the low and high extremes, respectively, in the wild-type distribution. This result suggests a criterion that an appropriate CAI_H_ threshold can be determined according to CAI_H_ of the wild-type sequences of genes to be expressed. Second, we compared the CAI_H_ thresholds of C1, C2, and C3 with the all endogenous genes in *R. erythropolis* (Fig. 5b). We found that C2 was moderately higher from the median of the endogenous gene distribution whereas C1 and C3 were lower and much higher than the median, respectively. This result suggests another criterion that CAI_H_ thresholds should be higher from the median of the endogenous gene distribution while avoiding extremely high values. Taken together, we recommend that the CAI_H_ threshold is selected so that it does not largely deviate from those of the wild-type sequences and the endogenous genes. We also note that although C2 was the best performing in our verification experiment, C1 and C3 achieved the similar performance to C2 (Fig. 4b). Their success rates were comparable: C2 (9 out of the 12 genes) versus C1 and C3 (8 out of the 12 genes). In addition, all of the 5 genes with poor wild-type expression levels were improved not only by C2, but also by C1 and C3. Therefore, the exact value of the CAI_H_ threshold may not drastically affect the performance of C-method as long as the above criteria are roughly satisfied.

## Discussion

We developed a codon optimization method to be used in *R. erythropolis*, an attractive host organism for recombinant protein expression. The method was developed based on the statistical analysis of our recombinant protein expression data from 204 genes. The resulting method named C-method was used to optimize the coding sequences of selected genes, achieving to increase protein expression levels compared with wild-type sequences. Our method will be a useful tool for improved expression of recombinant proteins in *R. erythropolis*, and possibly in other actinobacteria.

In the statistical analysis of recombinant protein expression data, our results on *R. erythropolis* were partly consistent with the previous study on *E. coli* (Fig. 1a; Supplementary Fig. S2). On the other hand, we observed the species-specific influence regarding the codon repetition rate and the amino acid repetition rate. Although the interpretation of this species specificity is not straightforward, we provide possible molecular mechanisms underlying this phenomenon. First, several studies reported an effect called codon order where the repeated use of the same type of codon increases translational efficiency^21,22^. These studies proposed a model that a ribosome can “recycle” a tRNA when scanning from a codon to the next codon if these neighboring codons correspond to the same type of tRNA, thereby enhancing translational efficiency. According to this model, codon repetition rates are expected to show positive correlation with protein expression levels. Second, some amino acid repeats such as proline-rich stretches are known to decrease translational efficiency by inducing ribosome stalling. (See the recent review^23^). This suggests that amino acid repetition rates negatively correlate with protein expression levels. Finally, and most importantly, the codon repetition rate and the amino acid repetition rate are not independent since codon repeats are necessarily translated into amino acid repeats. Therefore, the joint effect of these sequence features can be complicated. For example, increased codon repetition rates, which themselves imply higher protein expression levels, may result in lower protein expression levels due to the counter effect of increased amino acid repetition rates. In summary, we speculate that the species-specific influence of these sequence features may reflect a different balance between their effects in *R. erythropolis* and *E. coli* due to e.g. differences in the structures of ribosomal machineries and/or the copy numbers of tRNA genes. While the dissection of such possible causes cannot be performed in the present study, our results will contribute as an example showing the species-specific influence of sequence features on protein expression levels.

In the development of codon optimization methods, H-method based solely on the mRNA folding energy was first devised (Fig. 2), and then improved to C-method by incorporating the codon frequency (Fig. 4). This was motivated by the feedback from our experimental data that genes whose protein expression levels could not be increased by H-method contained rare codons (Fig. 3). Such a feedback strategy may also be useful for developing a codon optimization method for host organisms other than *R. erythropolis*, including bacteria, fungi, insects and mammals^1^.

In the verification experiment of C-method (Fig. 4), 9 of 12 tested genes (75%) achieved increased expression levels. Especially for 5 genes whose expression levels for wild-type sequences were small or not detectable, all of them were improved by codon optimization. On the other hand, the improvement was not observed for the remaining 3 genes. We note that the expression levels of these genes were relatively high even with wild-type sequences before codon optimization. Their expression scores in our dataset (Supplementary Data S1) were 3 (high) for 2 genes and 2 (medium) for one gene. Thus, one possibility is that the expression of these genes is already at near-optimal levels, and is difficult to be further improved by codon optimization.

We note that our method currently has the limitations as summarized below. First, since C-method only considers ΔG_UH_ and CAI_H_, the improvement of protein expression may be hindered by the involvement of other sequence features. For example, the modulation of CAI_H_ in C-method only considers 33 head nucleotides, which suggests a possibility that the improvement is hindered by the poor CAI in downstream nucleotides. We confirmed that this possibility did not apply to the genes used in the verification experiment of C-method (Supplementary Fig. S4). The overall CAI of the failed genes did not show substantial differences from that of the succeeded genes. Nevertheless, the success rate of our method may be improved by incorporating new sequence features in addition to ΔG_UH_ and CAI_H_. Recently, Cambray *et al*. has conducted an expression analysis in *E. coli* using 244,000 genes^24^. Even with such a large-scale analysis, they have reported that sequence features (similar to those used in our study) only explain approximately 30% of variations in protein expression levels, suggesting the existence of unknown sequence features. To explore such new sequence features, larger-scale analysis of recombinant protein expression in *R. erythropolis* as well as in other host organisms will be necessary. Second, our method was developed based upon sequence features influencing translational efficiency. Thus, the improvement of protein expression will be limited when the expression is hindered by factors other than translational efficiency. These factors include the membrane sorting, the S-S bond formation, and the toxicity of proteins to be expressed. We note that all proteins used in this study were cytoplasmic proteins not including transmembrane proteins nor proteins with S-S bonds (Supplementary Data S1). While this choice of proteins enabled us to develop and evaluate our method focusing on translational efficiency, the improvement based on the other factors was not addressed in the present study.

In the present study, we addressed the use of codon optimization methods mainly for increasing expression levels. On the other hand, there is also some need for decreasing expression levels, which includes weakening unnecessary fluxes in a metabolic pathway to improve the production of objective metabolites^25^. In this regard, our method may also be useful since the L-series sequences designed by the deoptimization of the mRNA folding energy succeeded to decrease expression levels for most of the tested genes (Fig. 2). Another issue not addressed in this study is a fine tuning of expression levels, that is, not only to simply maximize or minimize expression levels, but to regulate expression at a desired level^26^. The design of coding sequences for that purpose may be possible by choosing synonymous variants with moderate values of sequence features, rather than those with the maximum or the minimum value. These points should be addressed as a future direction.

## Methods

### Recombinant protein expression dataset

#### Gene expression

A total of 204 genes (listed in Supplementary Data S1) from *S. coelicolor* A3(2) was cloned into the inducible expression vector pTip-QC2^12^ at the NdeI restriction site (CATATG) in order to arrange the start codon at the same position. For genes whose start codon is other than ATG, the start codon was replaced with ATG. Each of the plasmid vector was introduced into the host strain, *R. erythropolis* L-88^27^. The recombinant strains were precultured in LB liquid media containing 34 µg/ml chloramphenicol at 28 °C for overnight. 2 ml of the preculture was transferred to 20 ml of the same fresh media containing 0.5 µg/ml thiostrepton and cultured with 120 rpm agitation for 16 hours. The cells were harvested and washed twice with 100 mM sodium phosphate buffer. The cell pellets were then resuspended with the same buffer containing 8 M urea and cell disruption was performed by glass beads using the Multi-beads shocker instrument (Yasui Kikai, Japan). The supernatant of the disrupted sample was used as the denatured crude cell extract.

#### Protein expression level measurement

20 µg of the crude cell extract protein from each of the recombinants was analyzed by SDS-PAGE and the protein was visualized by Coomassie Brilliant Blue staining. The expression level of each recombinant protein was manually scored and classified into 1-3 (1, low or not detected; 2, medium; 3, high) with visual inspection by bare eye (Supplementary Fig. S1; Supplementary Data S1).

### Sequence features and statistical analysis

CAI and tAI were calculated as previously described^17,18^ where the genomic codon usage in *R. erythropolis* was obtained from Codon Usage Database^28^ with the accession number 234621. For tAI, the weight matrix (also known as the codon-tRNA interaction matrix) was calculated by the method proposed in the previous study^29^. Briefly, we adjusted the weight matrix considering the copy numbers of tRNA genes and the codon usage bias in the *R. erythropolis* genome. The resulting weight matrix is available at the authors’ GitHub web site^30^. The codon repetition rate^9^ was calculated by ${\sum }_{i=1}^{L}{d}_{i}^{-1}/L$ where *d*_*i*_ is the distance from each codon position to the next occurring position of the same type of codon, and *L* is the sequence length. The amino acid repetition rate^9^ was calculated similarly except that the repetition was counted for the type of encoded amino acids rather than the type of codons. ΔG_UH_ was calculated by EnsembleEnergy program version 5.8.1 in RNAstructure package^19^. For statistical analysis, polyserial correlation coefficients^20^ and their p-values were calculated using polycor package version 0.7.9 in R software^31^. CAI_H_ was calculated similarly to CAI focusing on 33 head nucleotides (i.e. 11 codons). Rare codons were defined as codons with normalized codon frequencies less than 0.1. For each type of codon *c*, a normalized codon frequency was defined as *w*_c_ = *f*_*c*_/max_*s*_ *f*_*s*_ where *f*. is a genomic codon frequency and *s* is a synonymous codon encoding the same amino acid as *c*.

### Codon optimization methods

The algorithms of H-method and C-method are described in Results. The software is available at the authors’ GitHub web site^30^. Coding sequences designed by our method are shown in Supplementary Data S3.

### Verification experiments

PCR amplification and cloning of the codon-optimized genes: The nucleotide sequences of the codon-optimized genes were listed in Supplementary Data S3. The designed genes were amplified by PCR using the wild-type genes as the templates and the forward primers containing the codon-altered nucleotide sequences. The both forward and reverse PCR primers also contained overhang sequences of the cloning vector, pTip-QC2, to combine them by In-Fusion cloning (TAKARA, Japan). The PCR primers used in this study were listed in Supplementary Data S4. The resultant plasmids were introduced into the host strain, and the protein expression experiment was performed as described above except for the culture volume of 11 ml and 10 µg of the crude extract protein used for the SDS-PAGE analysis.

#### Measurement of protein expression levels

The SDS-PAGE gels were optically analyzed by the image analyzer WSE-6100 LuminoGraph (ATTO, Japan). Quantitative analysis of the recombinant protein was performed by the ImageSaver6 / CS Analyzer software (ATTO, Japan). The expression level of each mutant relative to the wild type was determined by the average value of three independent recombinant clones.

## Supplementary information

Supplementary Information

Supplementary Data S1

Supplementary Data S2

Supplementary Data S3

Supplementary Data S4

Supplementary Data S5

## Data Availability

All data in this study are available in the main text or in Supplementary Material. The program implementation of our codon optimization methods is available in the authors’ GitHub web site^30^.
