# Supplementary material for: Developing a codon optimization method for improved expression of recombinant proteins in actinobacteria
Source: Sci Rep. 2019 Jun 6;9:8338. doi: 10.1038/s41598-019-44500-z (PMC6554278; doi:10.1038/s41598-019-44500-z)
Supplement: Supplementary file 1 — Supplementary Information [file 41598_2019_44500_MOESM1_ESM.pdf]

Supplementary Information

Developing a codon optimization method for improved expression of recombinant proteins in actinobacteria

Yutaka Saito[1,2]†, Wataru Kitagawa[3,4]†, Toshitaka Kumagai[5], Naoyuki Tajima[1], Yoshiyuki Nishimiya[3], Koichi Tamano[3], Yoshiaki Yasutake[3], Tomohiro Tamura[3,4]*, Tomoshi Kameda[1]*

1 Artificial Intelligence Research Center, National Institute of Advanced Industrial Science and Technology (AIST), 2-4-7 Aomi, Koto-ku, Tokyo 135-0064, Japan.

2 Computational Bio Big-Data Open Innovation Laboratory (CBBD-OIL), National Institute of Advanced Industrial Science and Technology (AIST), 3-4-1 Okubo, Shinjuku-ku, Tokyo 169-8555, Japan.

3 Bioproduction Research Institute, National Institute of Advanced Industrial Science and Technology (AIST), 2-17-2-1 Tsukisamu-Higashi, Toyohira-ku, Sapporo 062-8517, Japan.

4 Graduate School of Agriculture, Hokkaido University, Kita 9-Nishi 9, Kita-ku, Sapporo 060-8589, Japan.

5 Fermlab Inc., 913, 4-3-1 Shirakawa, Koto-ku, Tokyo 135-0021, Japan.

† Joint first authors

* Joint corresponding authors

* Correspondence to Tomohiro Tamura (t-tamura@aist.go.jp); Tomoshi Kameda (kameda-tomoshi@aist.go.jp)

Legends for Supplementary Figures and Supplementary Data

Supplementary Fig. S1. Examples of SDS-PAGE gels and integer expression scores. For each of integer expression scores 1 (a), 2 (b), and 3 (c), three examples of SDS-PAGE gels are shown. The expected positions of the recombinant proteins are indicated by black arrows. +: samples with protein expression induced. -: negative control where the induction of expression was not performed. The figure is included in this file.

Supplementary Fig. S2. Influence of sequence features in *R. erythropolis* compared with *E. coli.* (a) *R. erythropolis*. For each type of sequence feature, a polyserial correlation coefficient between feature values and expression levels is shown with its p-value. The same figure as in Fig. 1a. (b) *E. coli*. The results taken from the previous study [9] are shown in a similar way to (a). The z-scores of logistic regression coefficients represent the correlation of feature values and expression levels, similarly to the polyserial correlation coefficients in our study. Note that the p-values tend to be smaller for *E. coli* compared with *R. erythropolis*, partly due to the larger number of genes used in the study: *E. coli* (6348 genes) versus *R. erythropolis* (204 genes). The figure is included in this file.

Supplementary Fig. S3. ΔG_{UH} and CAI_H for wild-type and optimized sequences. For visualization, ΔG_{UH} is represented as the standard score $50 + {}^{10(\Delta G_{UH} - \mu)}/_{\sigma}$ where μ

and σ are the mean and the standard deviation, respectively, computed from all possible synonymous variants regarding 33 head nucleotides. The figure is included in this file.

Supplementary Fig. S4. Failure of C-method cannot be explained by poor codon frequencies in the entire coding sequences. For each of the C1, C2, and C3 sequences, CAI (a, c, e) and the rare codon count (b, d, f) of the entire coding sequences are compared between genes whose expression levels were increased by C-method (indicated as "Increased" in Fig. 4b) or not (indicated as "Unchanged" or "Decreased" in Fig. 4b). n.s.: $p > 0.05$ with Mann-Whitney *U* test. The figure is included in this file.

Supplementary Data S1. Recombinant protein expression levels and sequence feature values for the 204 genes. The data are available as a separate Excel file.

Supplementary Data S2. Rare codon counts in the H-series sequences at 33 head nucleotides. The data are available as a separate Excel file.

Supplementary Data S3. Wild-type and designed coding sequences for the selected 12 genes. The data are available as a separate Excel file.

Supplementary Data S4. Forward (-F) and reverse (-R) primers used in this study. Reverse primers were used for the amplification of both wild-type and variant genes. The data are available as a separate Excel file.

Supplementary Data S5. Raw image data of SDS-PAGE used in Figs. 2 and 4. The data are available as a separate zip file.

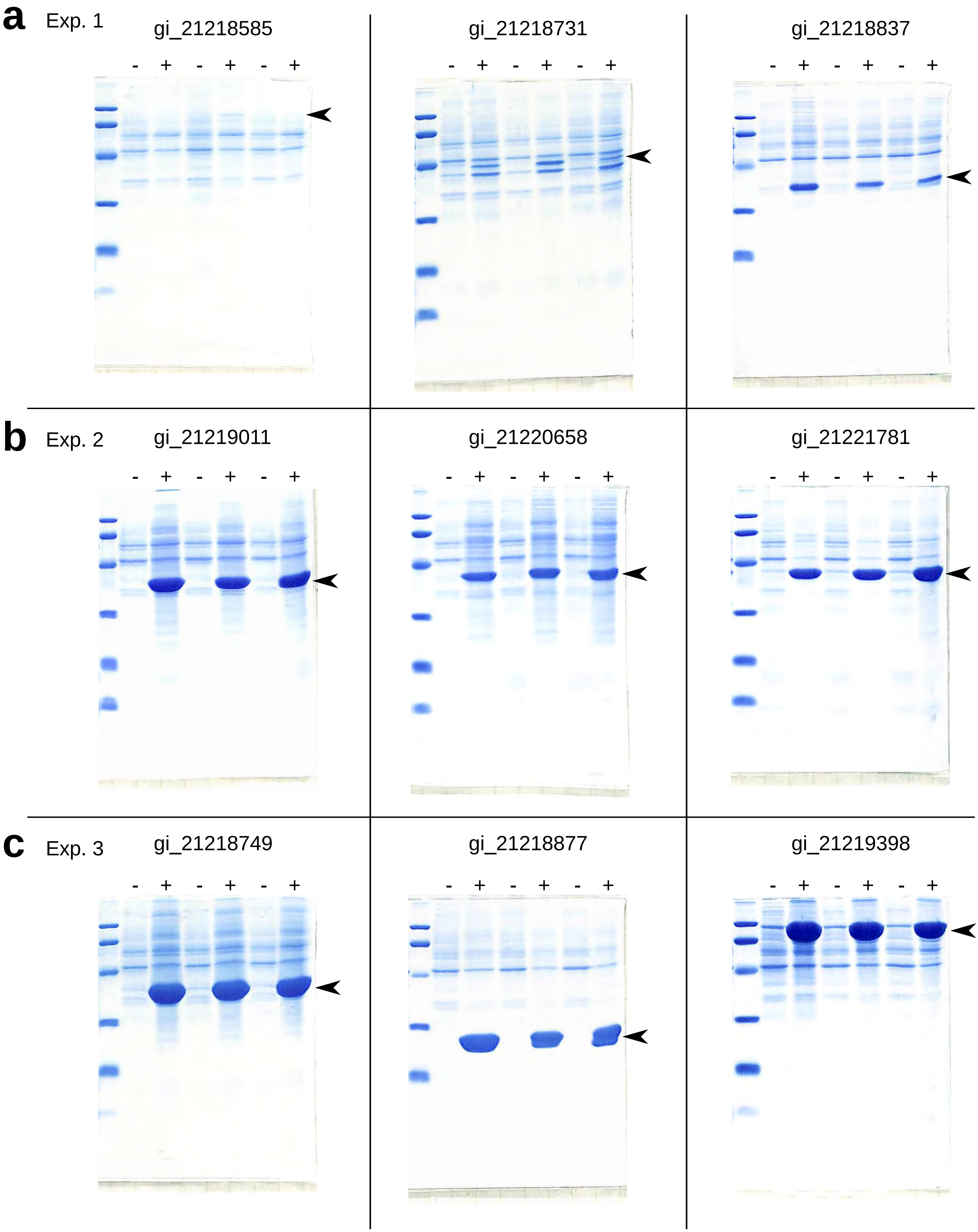

Supplementary Fig. S1

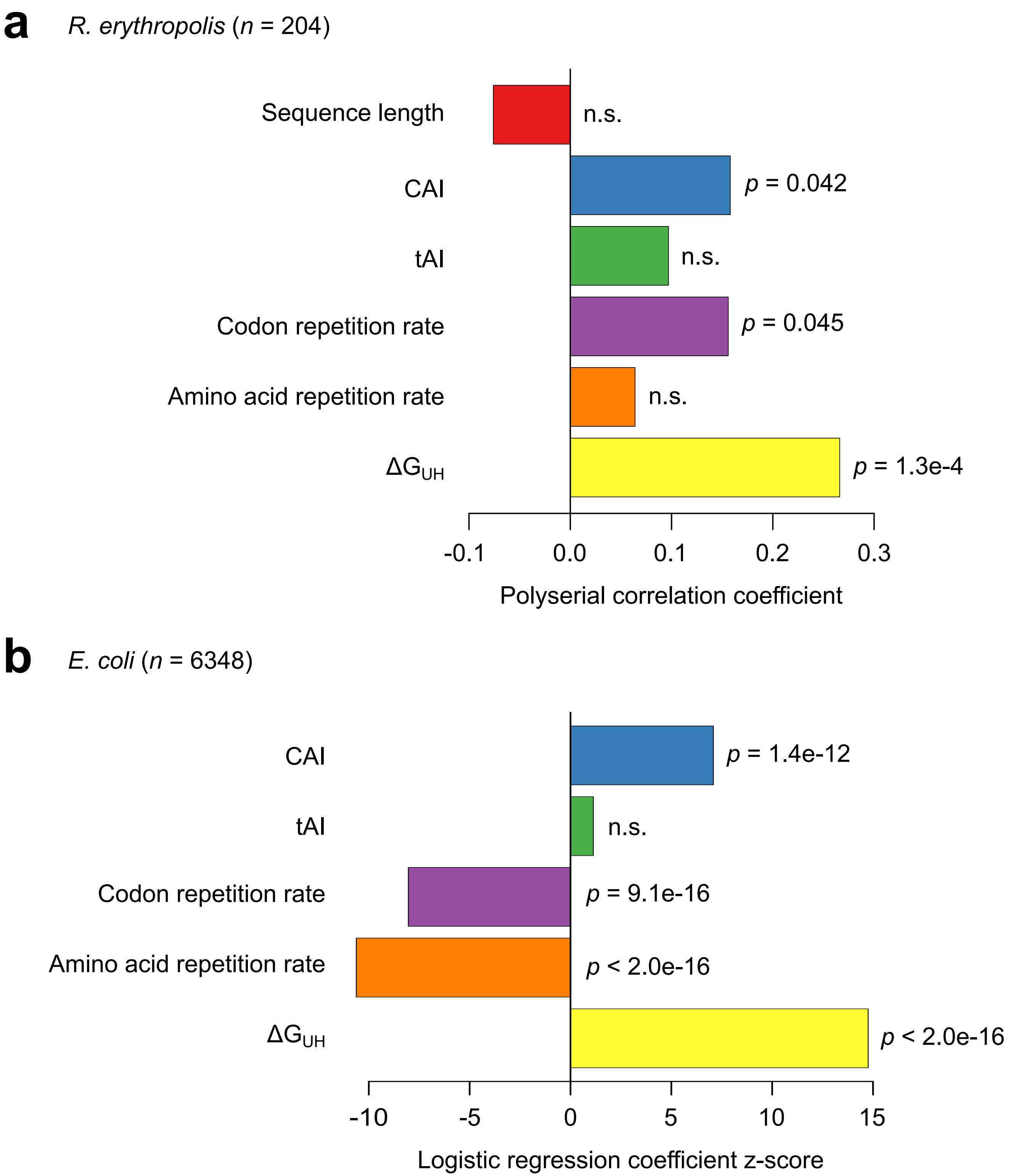

Supplementary Fig. S2

a

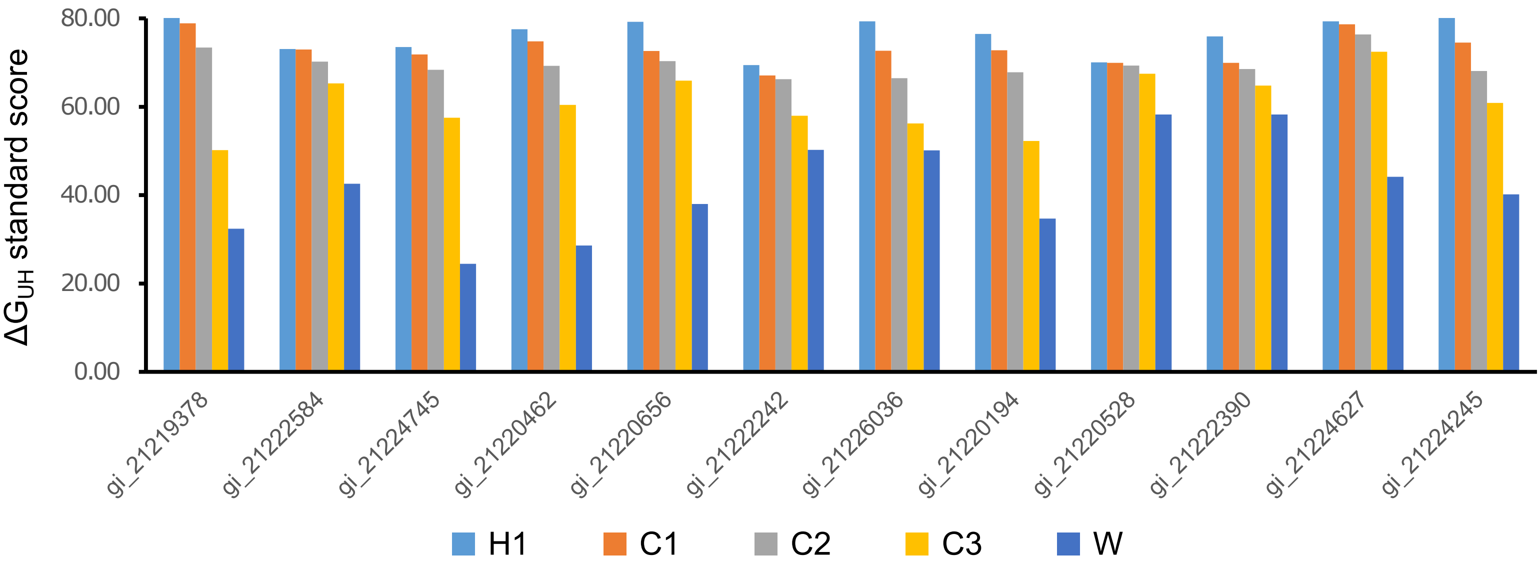

b

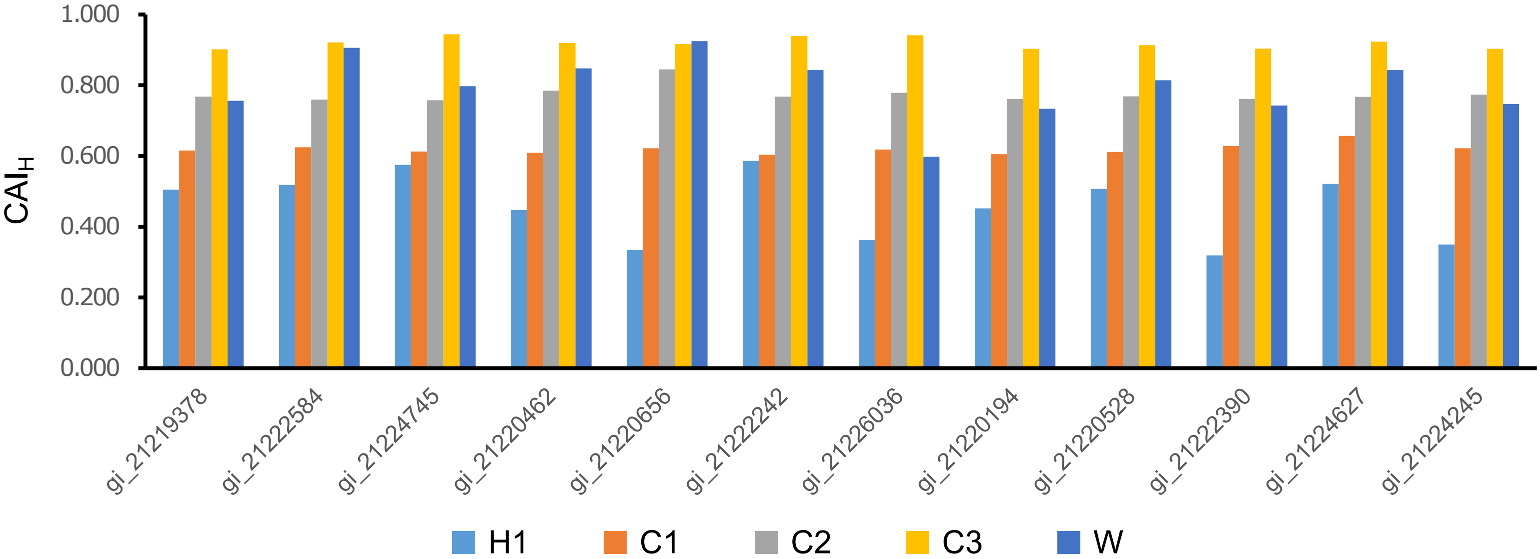

Supplementary Fig. S3

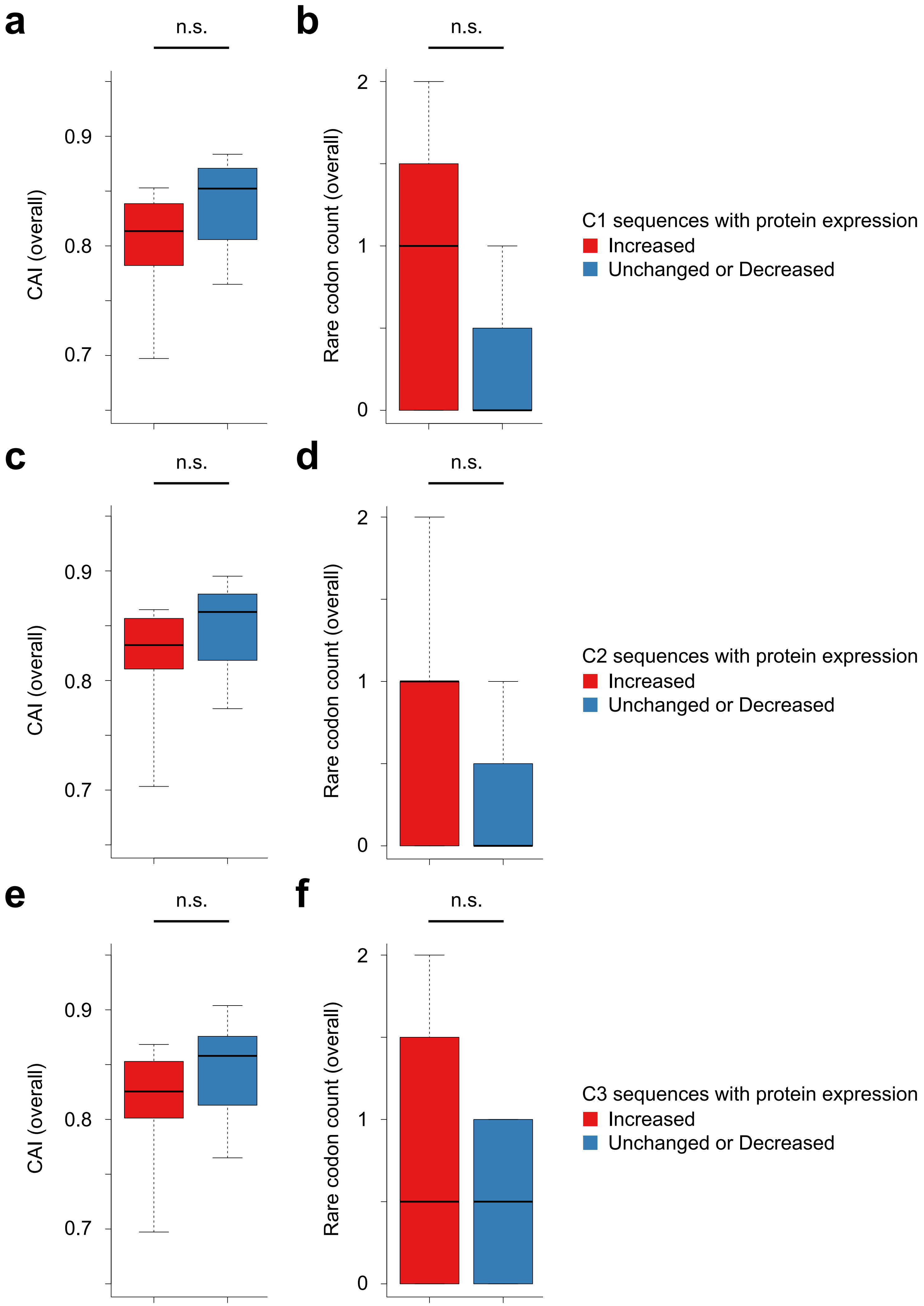

Supplementary Fig. S4
