# Supplementary figures and images for: Developing a codon optimization method for improved expression of recombinant proteins in actinobacteria
Source: Sci Rep. 2019 Jun 6;9:8338. doi: 10.1038/s41598-019-44500-z (PMC6554278; doi:10.1038/s41598-019-44500-z)

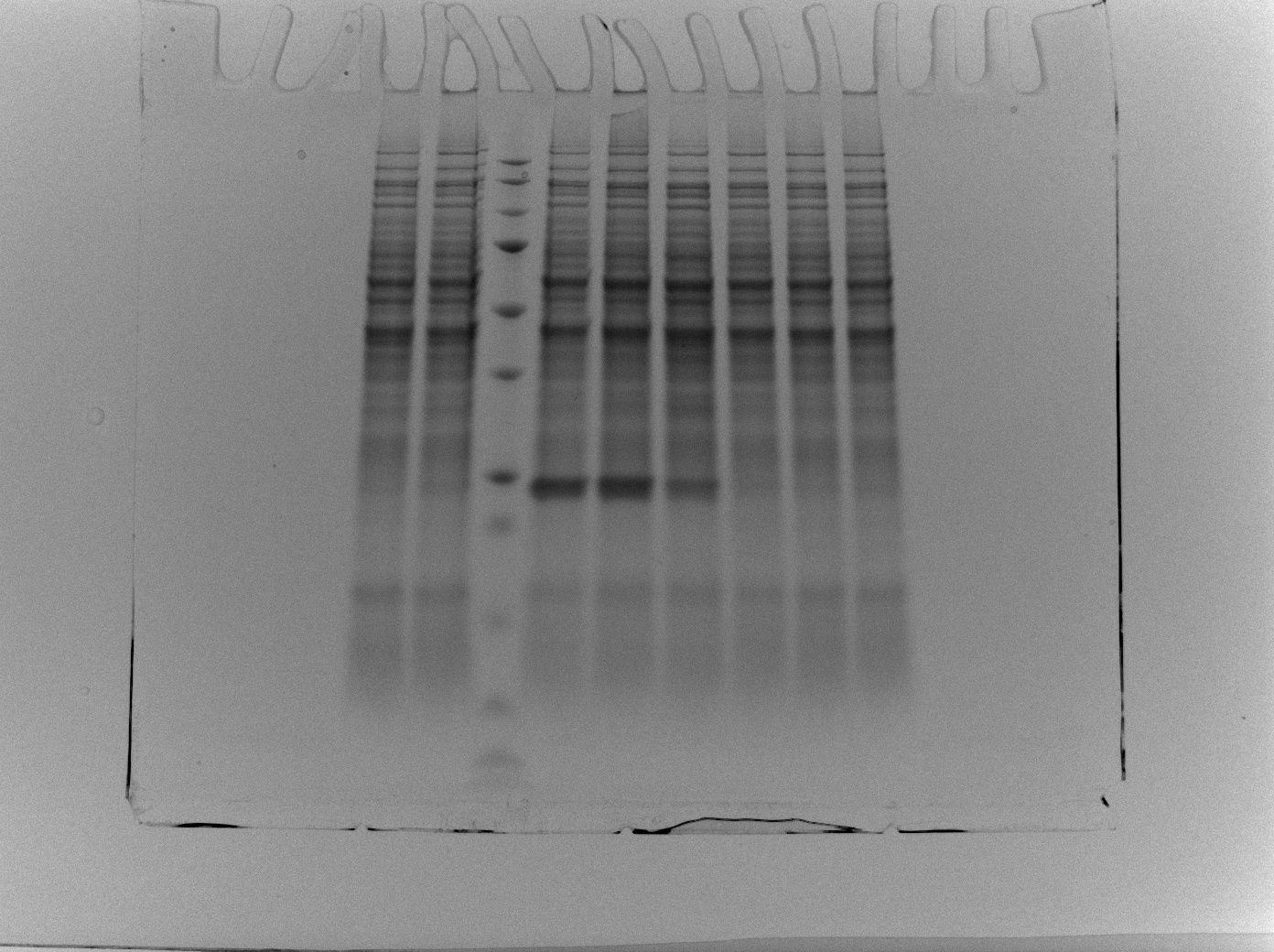

Supplement: Supplementary file 6 — Supplementary Data S5 [file 41598_2019_44500_MOESM6_ESM.zip › D2_SupDataS5/Fig2_raw_image/01_21229378_HL.jpg]

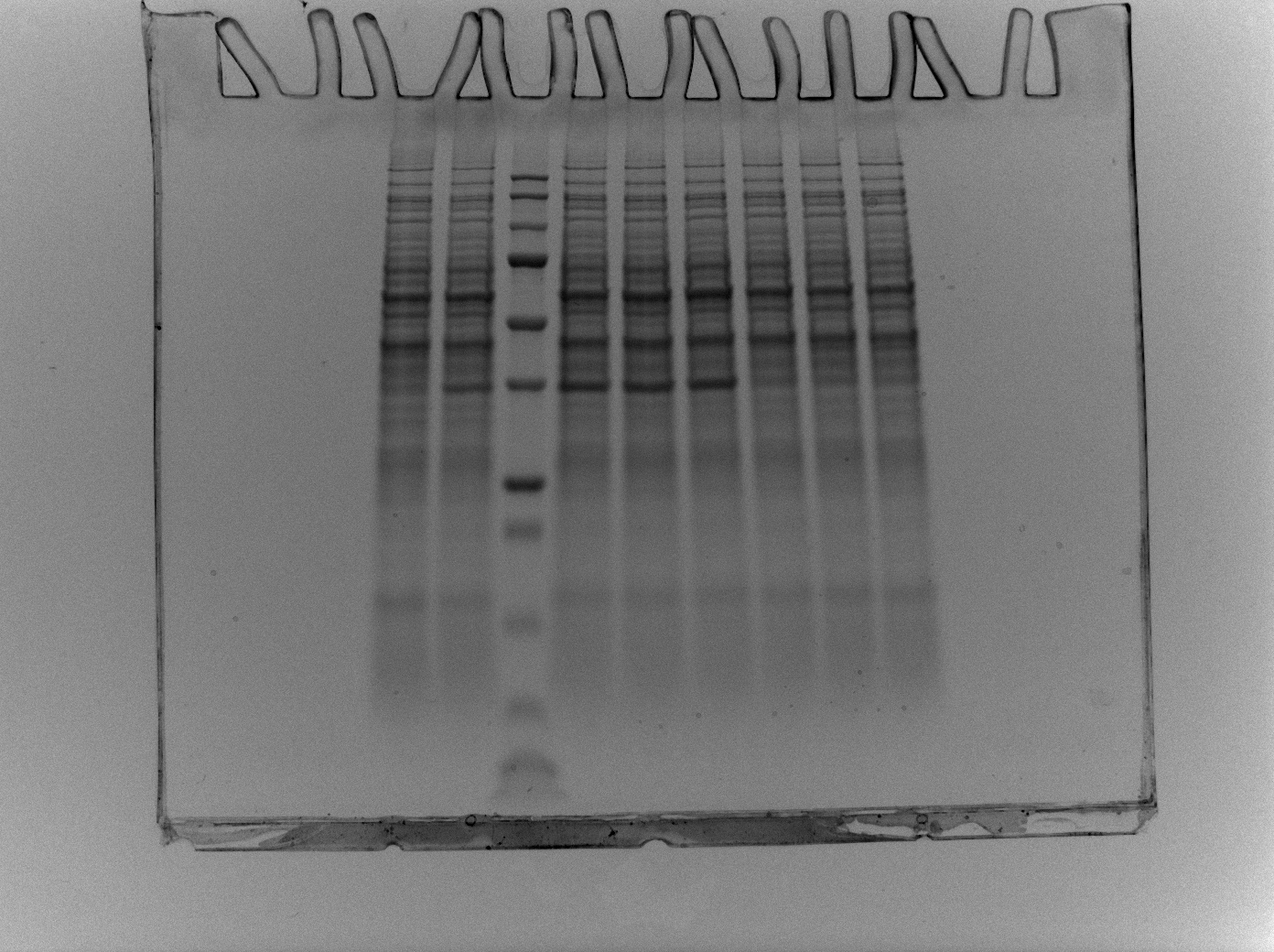

Supplement: Supplementary file 6 — Supplementary Data S5 [file 41598_2019_44500_MOESM6_ESM.zip › D2_SupDataS5/Fig2_raw_image/02_21222584_HL.jpg]

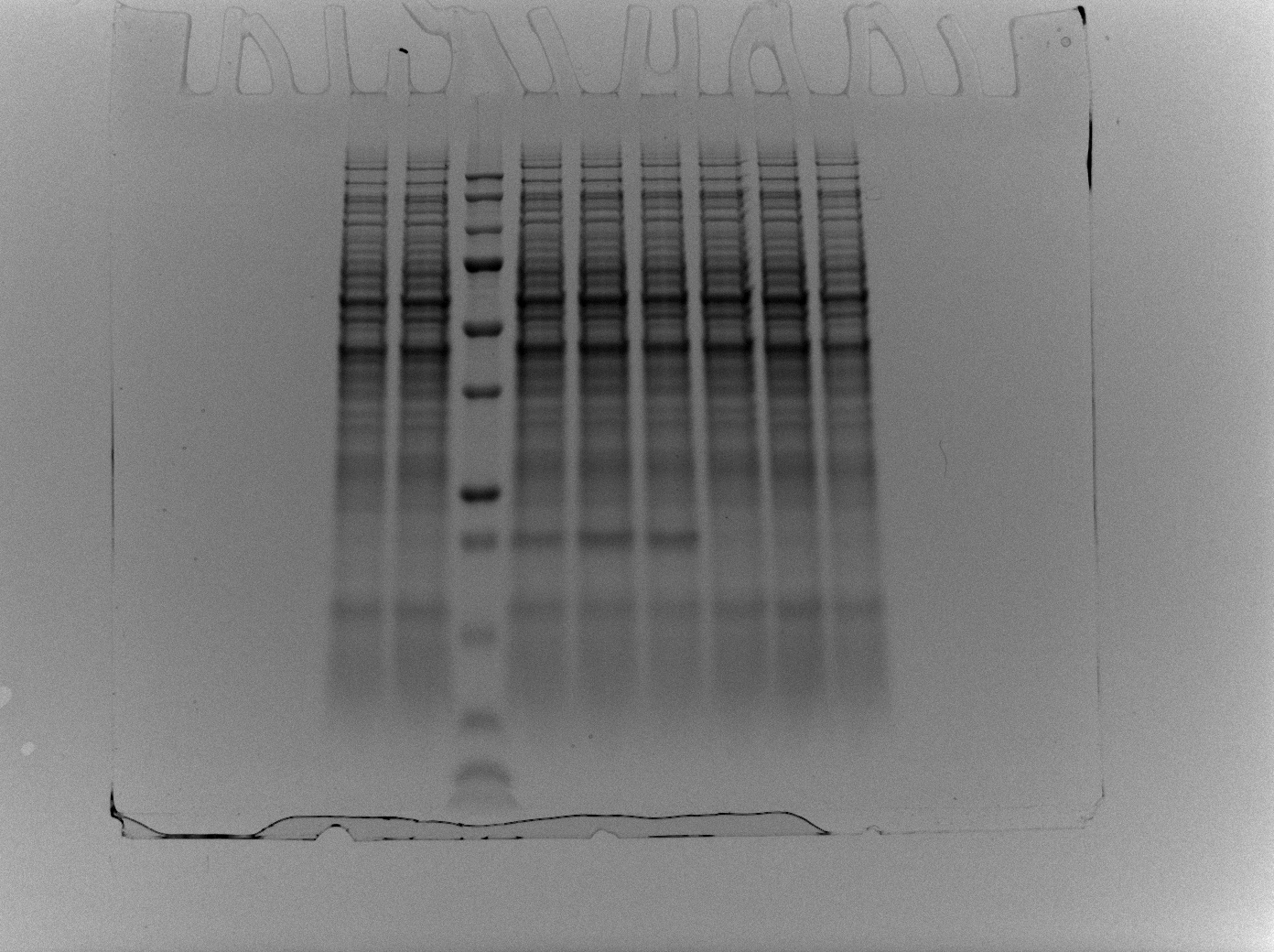

Supplement: Supplementary file 6 — Supplementary Data S5 [file 41598_2019_44500_MOESM6_ESM.zip › D2_SupDataS5/Fig2_raw_image/03_21224745_HL.jpg]

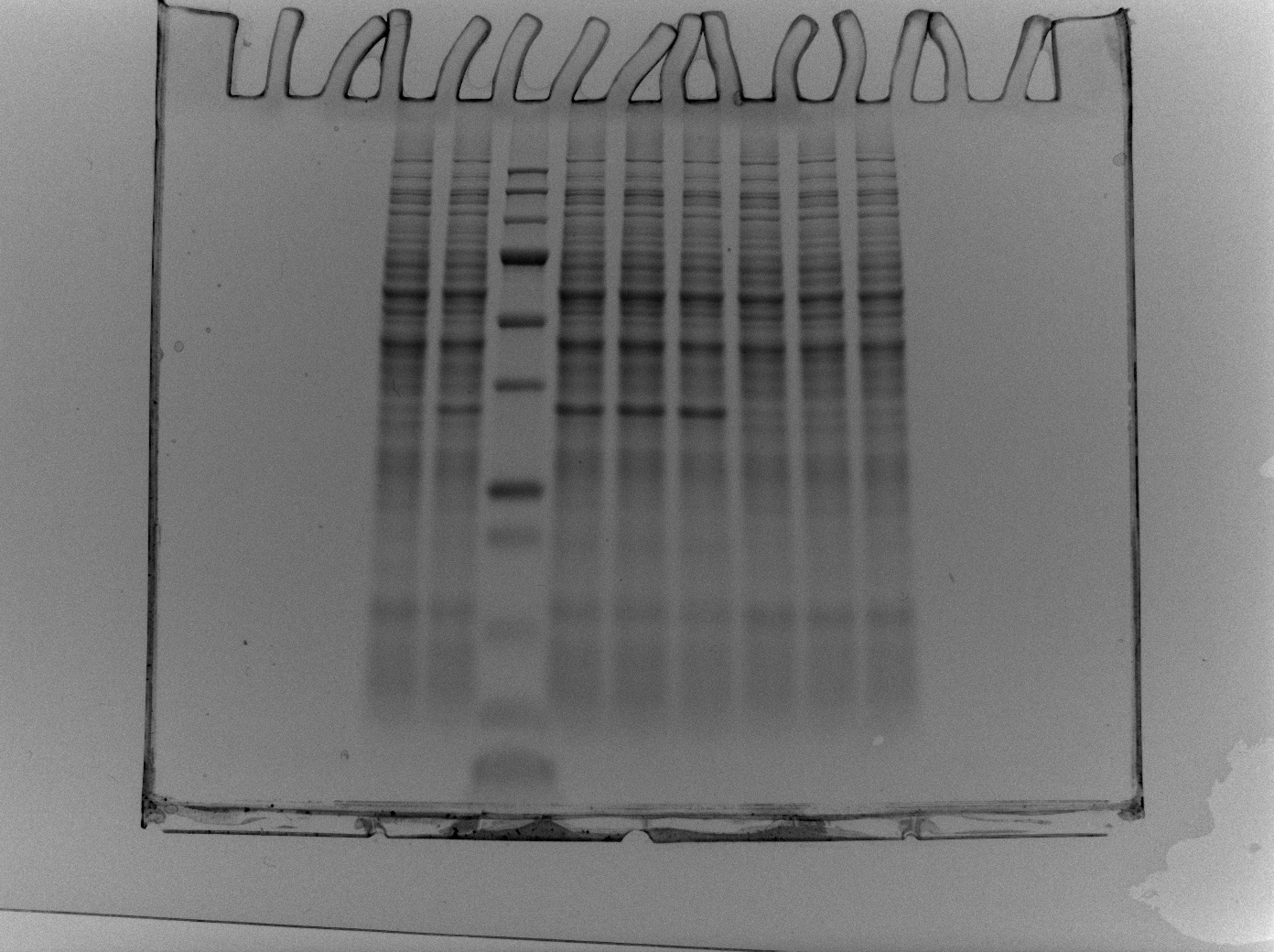

Supplement: Supplementary file 6 — Supplementary Data S5 [file 41598_2019_44500_MOESM6_ESM.zip › D2_SupDataS5/Fig2_raw_image/04_21220462_HL.jpg]

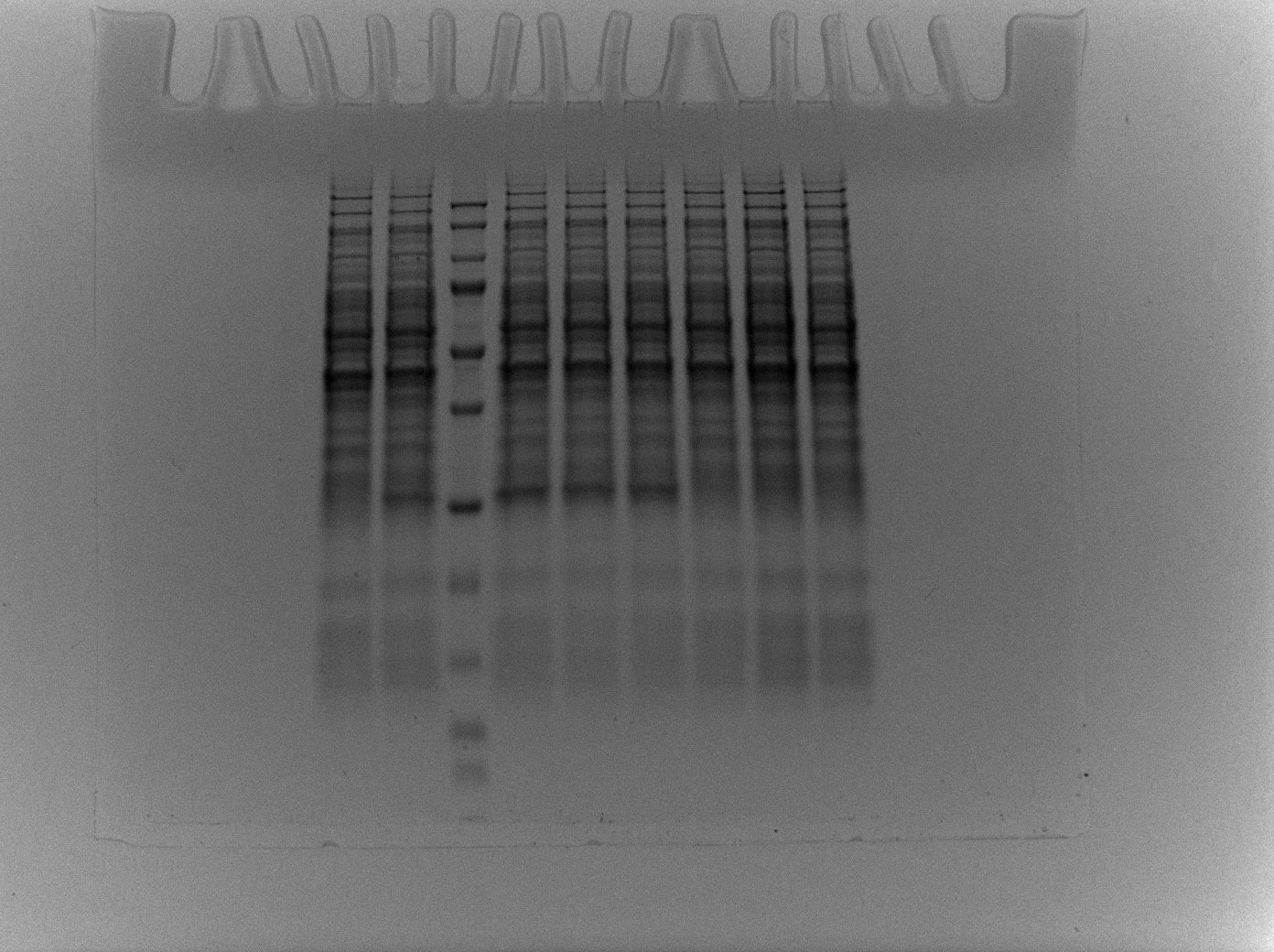

Supplement: Supplementary file 6 — Supplementary Data S5 [file 41598_2019_44500_MOESM6_ESM.zip › D2_SupDataS5/Fig2_raw_image/05_21220656_HL.jpg]

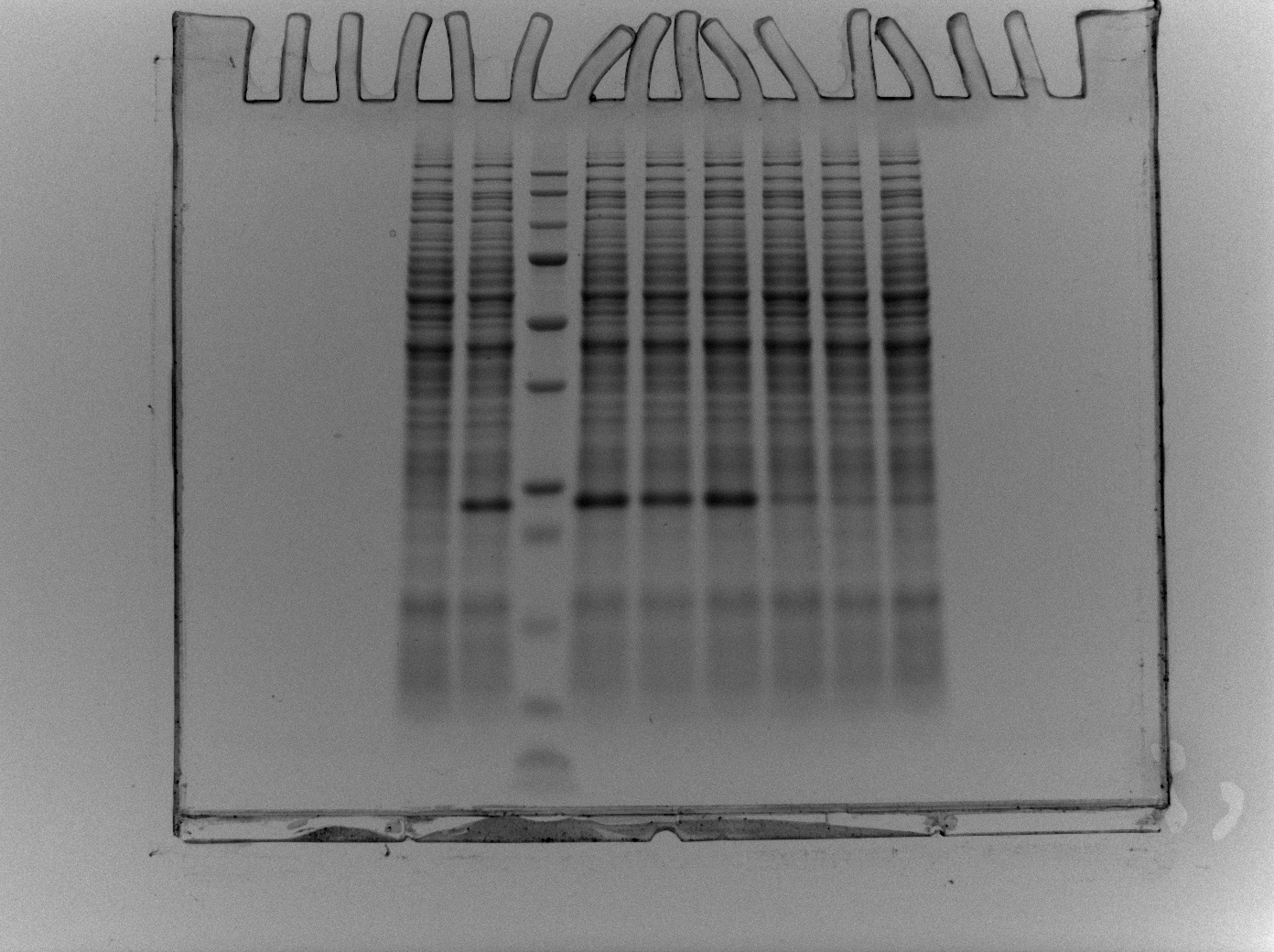

Supplement: Supplementary file 6 — Supplementary Data S5 [file 41598_2019_44500_MOESM6_ESM.zip › D2_SupDataS5/Fig2_raw_image/06_21222242_HL.jpg]

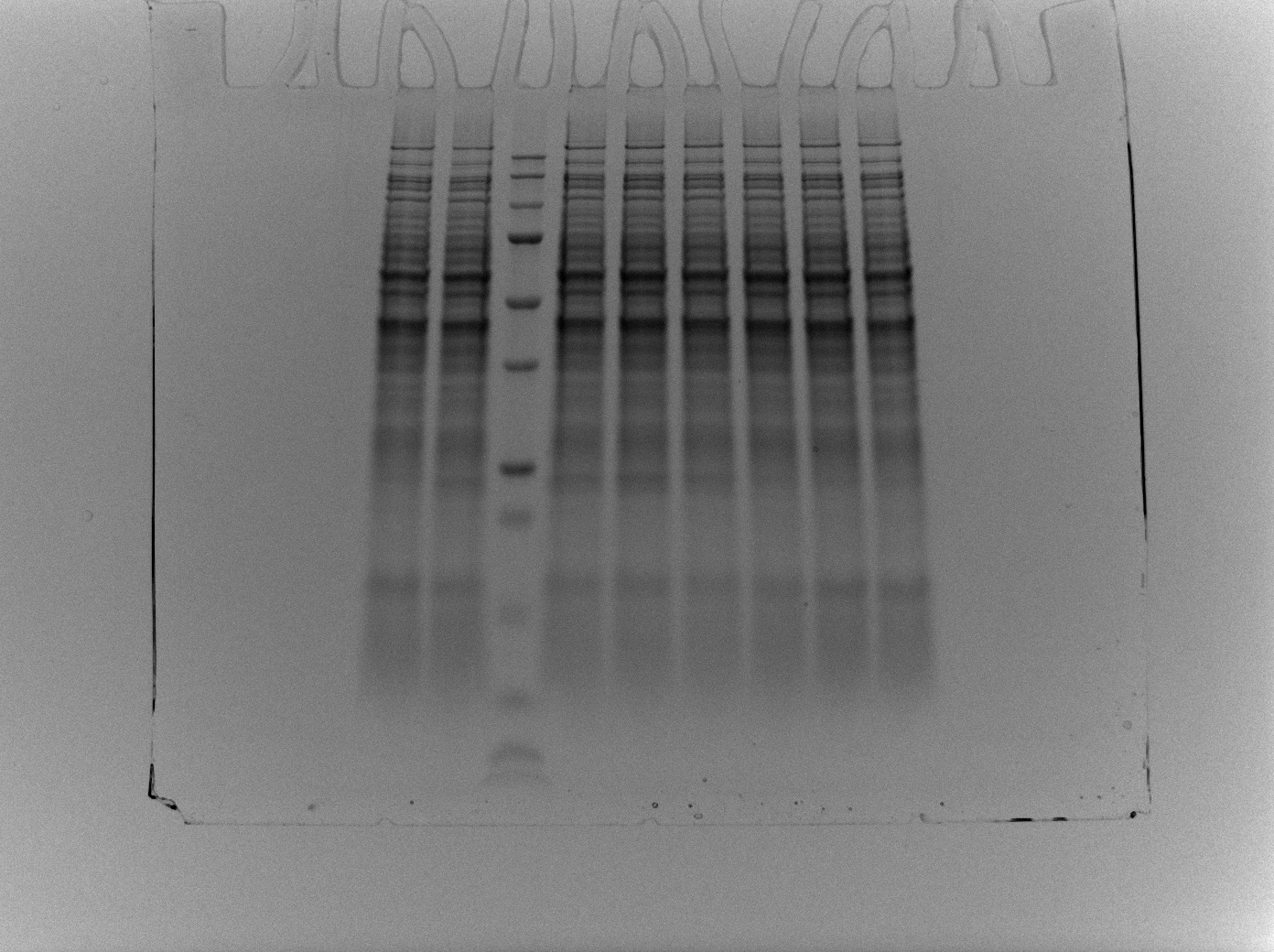

Supplement: Supplementary file 6 — Supplementary Data S5 [file 41598_2019_44500_MOESM6_ESM.zip › D2_SupDataS5/Fig2_raw_image/07_21226036_HL.jpg]

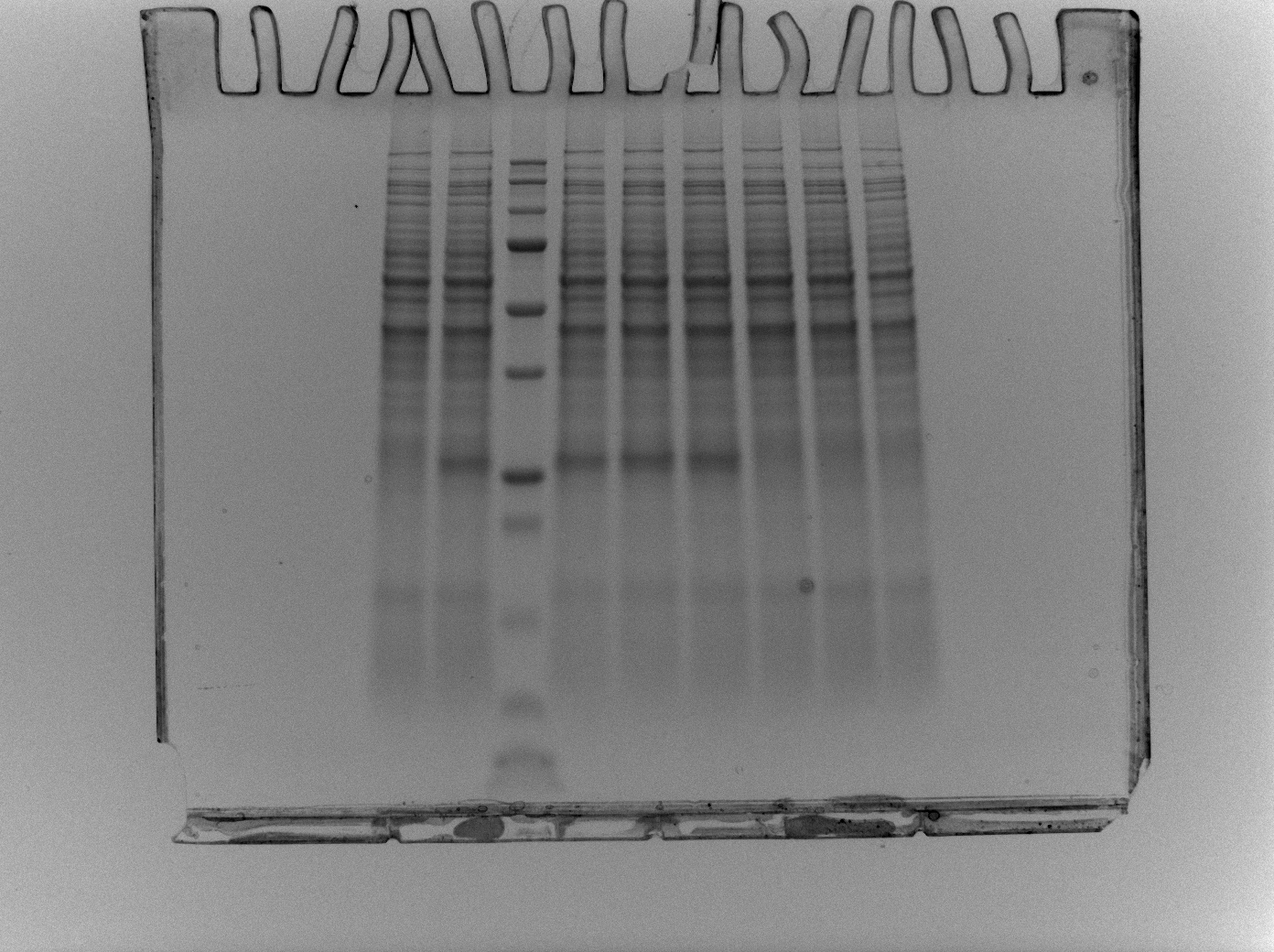

Supplement: Supplementary file 6 — Supplementary Data S5 [file 41598_2019_44500_MOESM6_ESM.zip › D2_SupDataS5/Fig2_raw_image/08_21220194_HL.jpg]

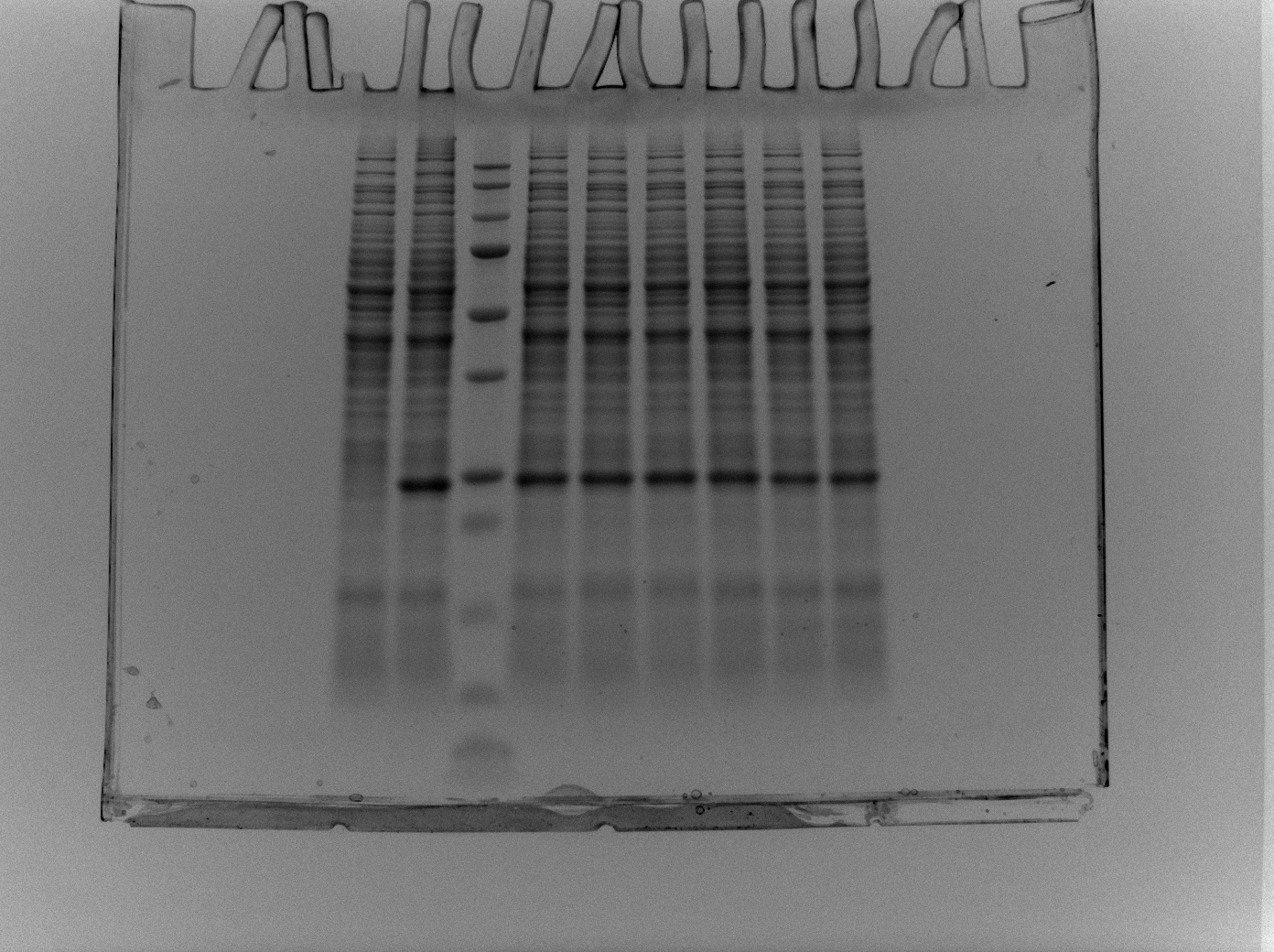

Supplement: Supplementary file 6 — Supplementary Data S5 [file 41598_2019_44500_MOESM6_ESM.zip › D2_SupDataS5/Fig2_raw_image/09_21220528_HL.jpg]

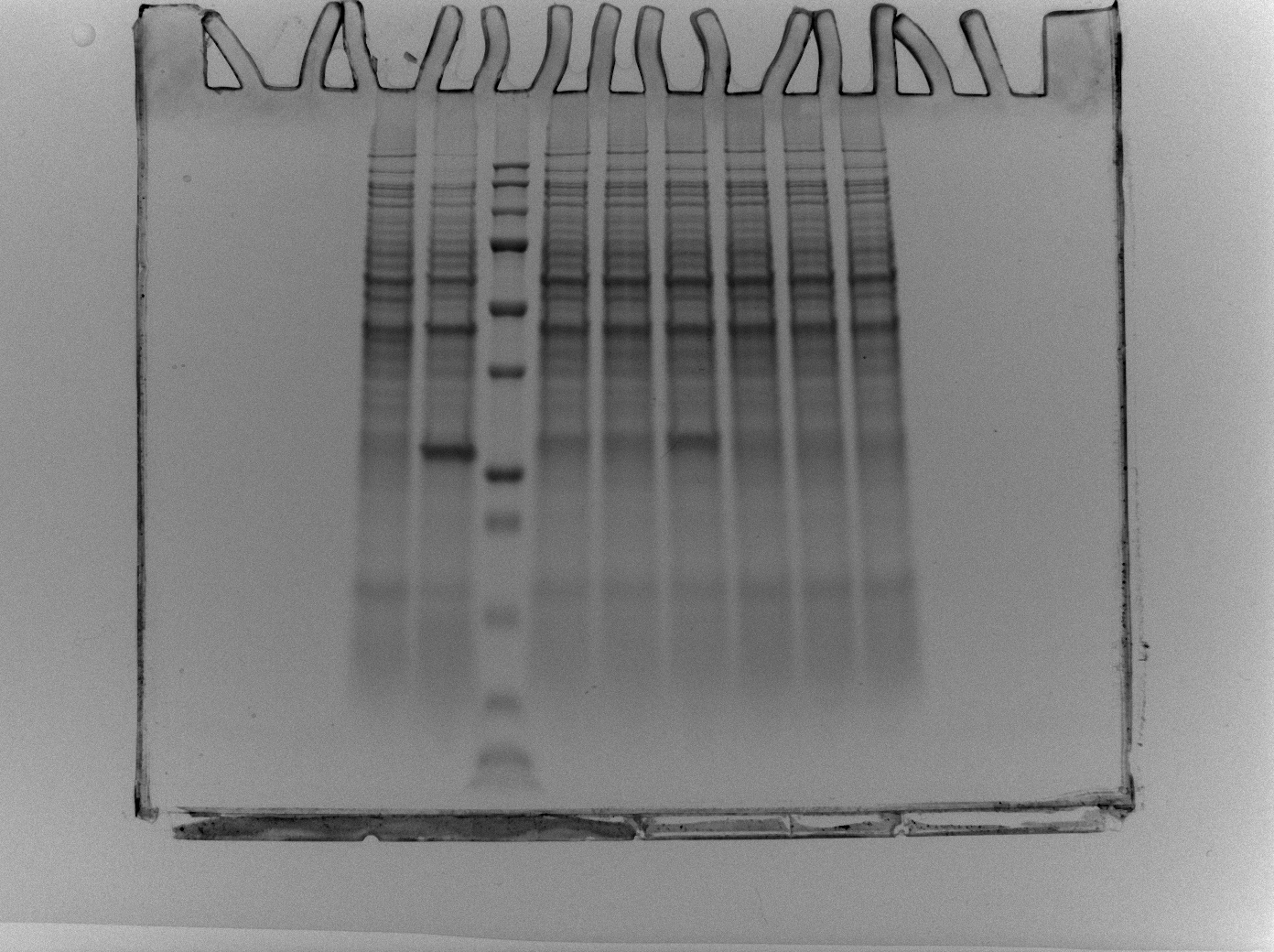

Supplement: Supplementary file 6 — Supplementary Data S5 [file 41598_2019_44500_MOESM6_ESM.zip › D2_SupDataS5/Fig2_raw_image/10_21222390_HL.jpg]

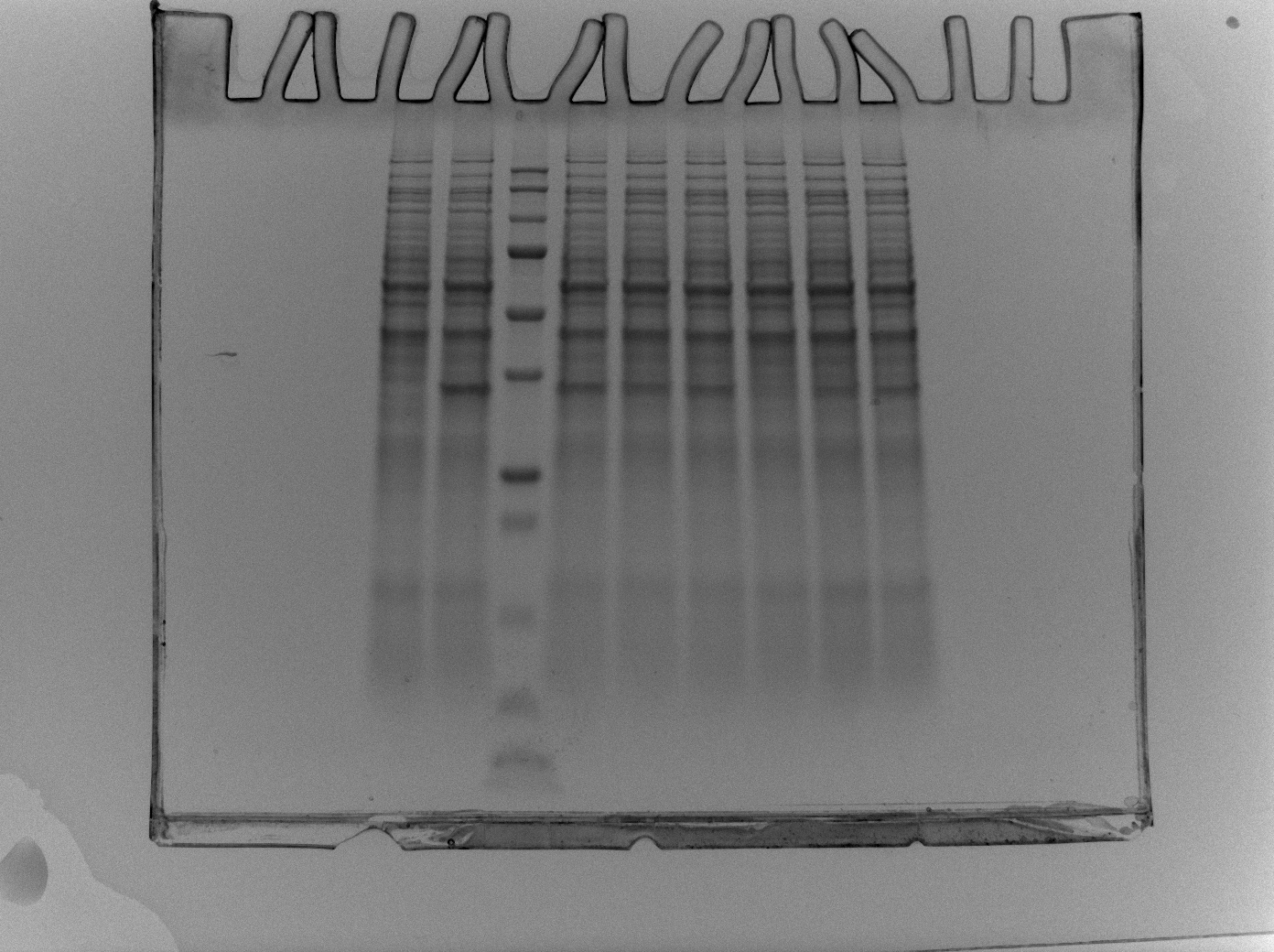

Supplement: Supplementary file 6 — Supplementary Data S5 [file 41598_2019_44500_MOESM6_ESM.zip › D2_SupDataS5/Fig2_raw_image/11_21224627_HL.jpg]

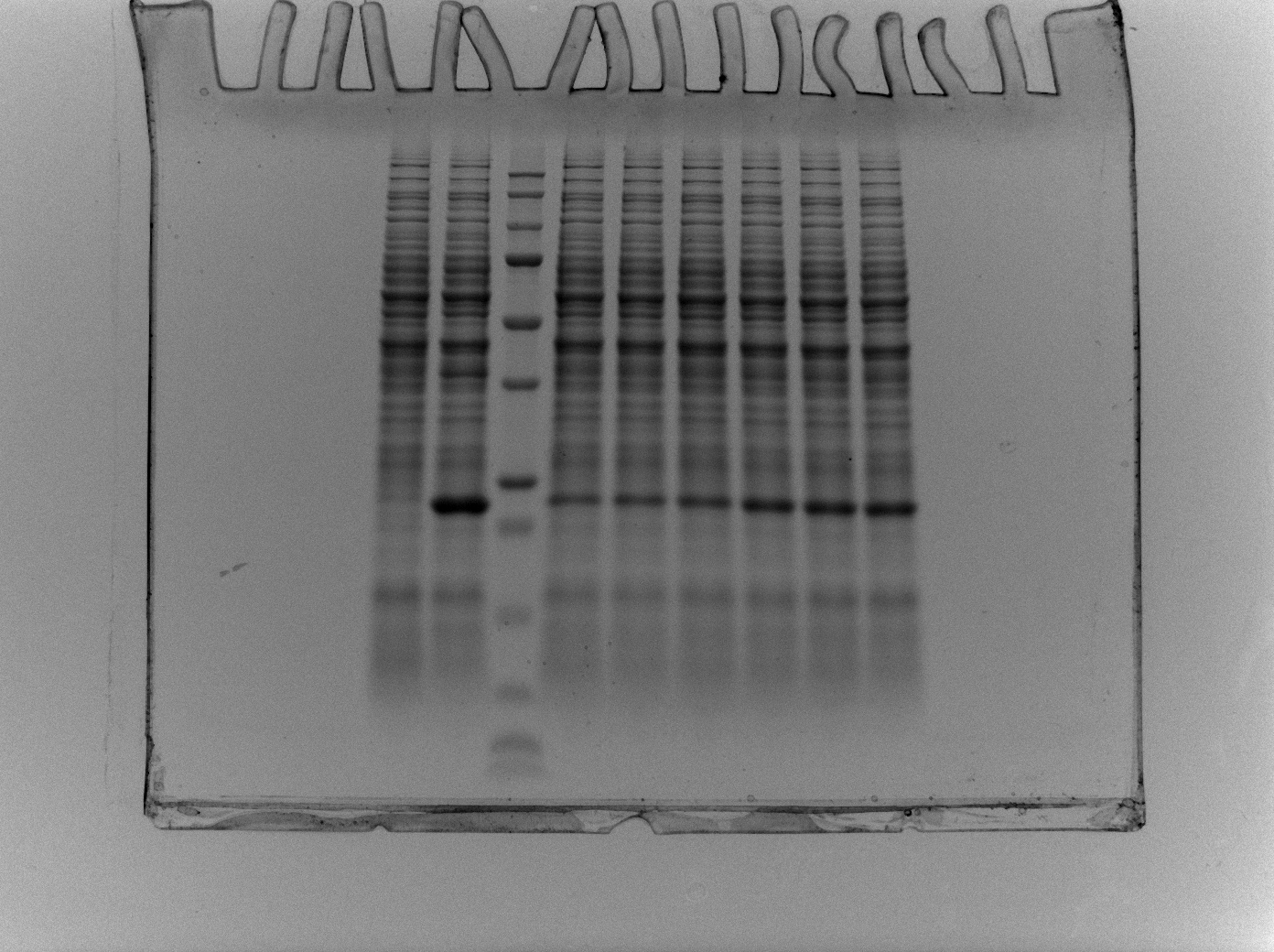

Supplement: Supplementary file 6 — Supplementary Data S5 [file 41598_2019_44500_MOESM6_ESM.zip › D2_SupDataS5/Fig2_raw_image/12_21224245_HL.jpg]

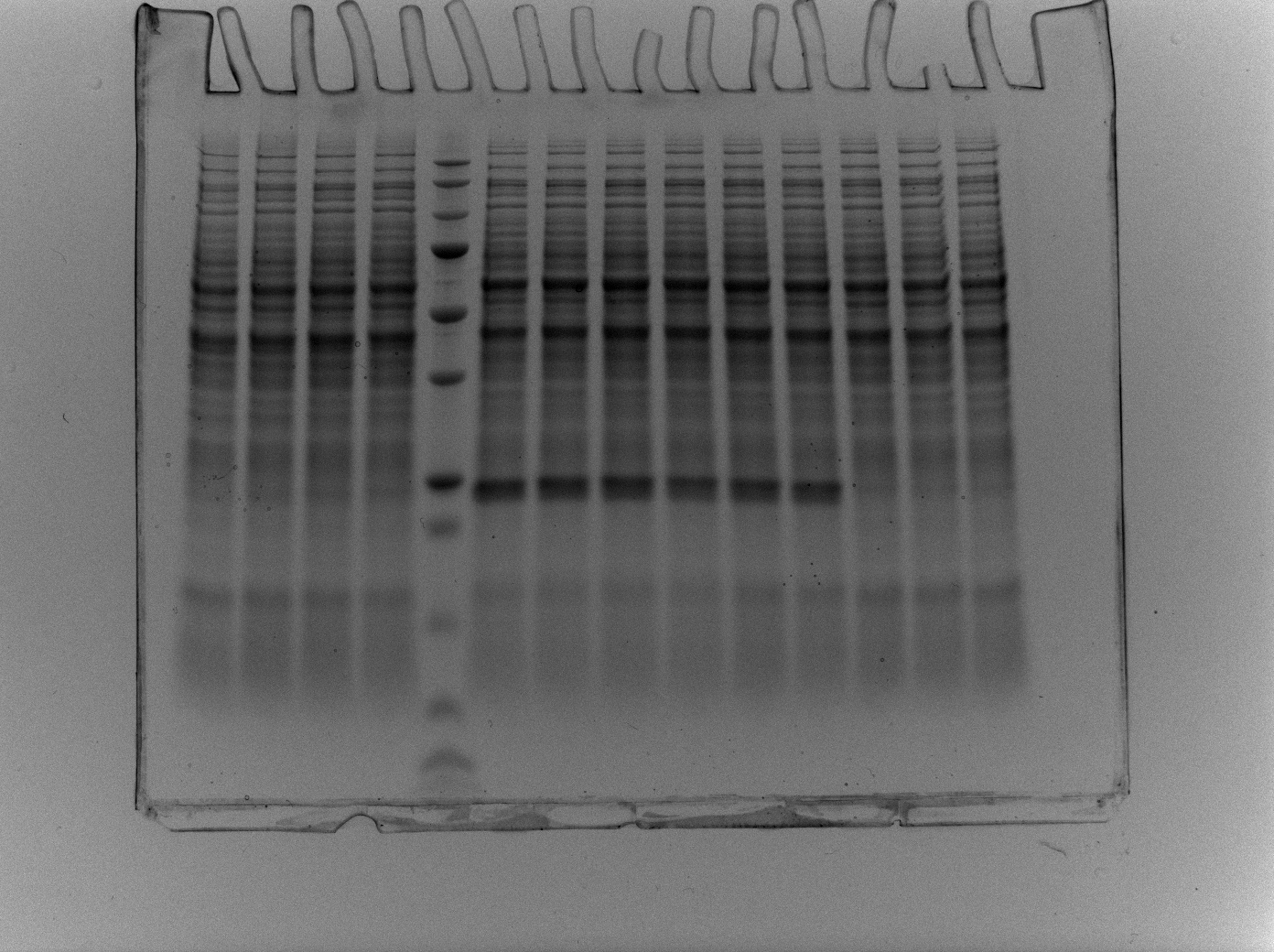

Supplement: Supplementary file 6 — Supplementary Data S5 [file 41598_2019_44500_MOESM6_ESM.zip › D2_SupDataS5/Fig4_raw_image/01_21229378_C.jpg]

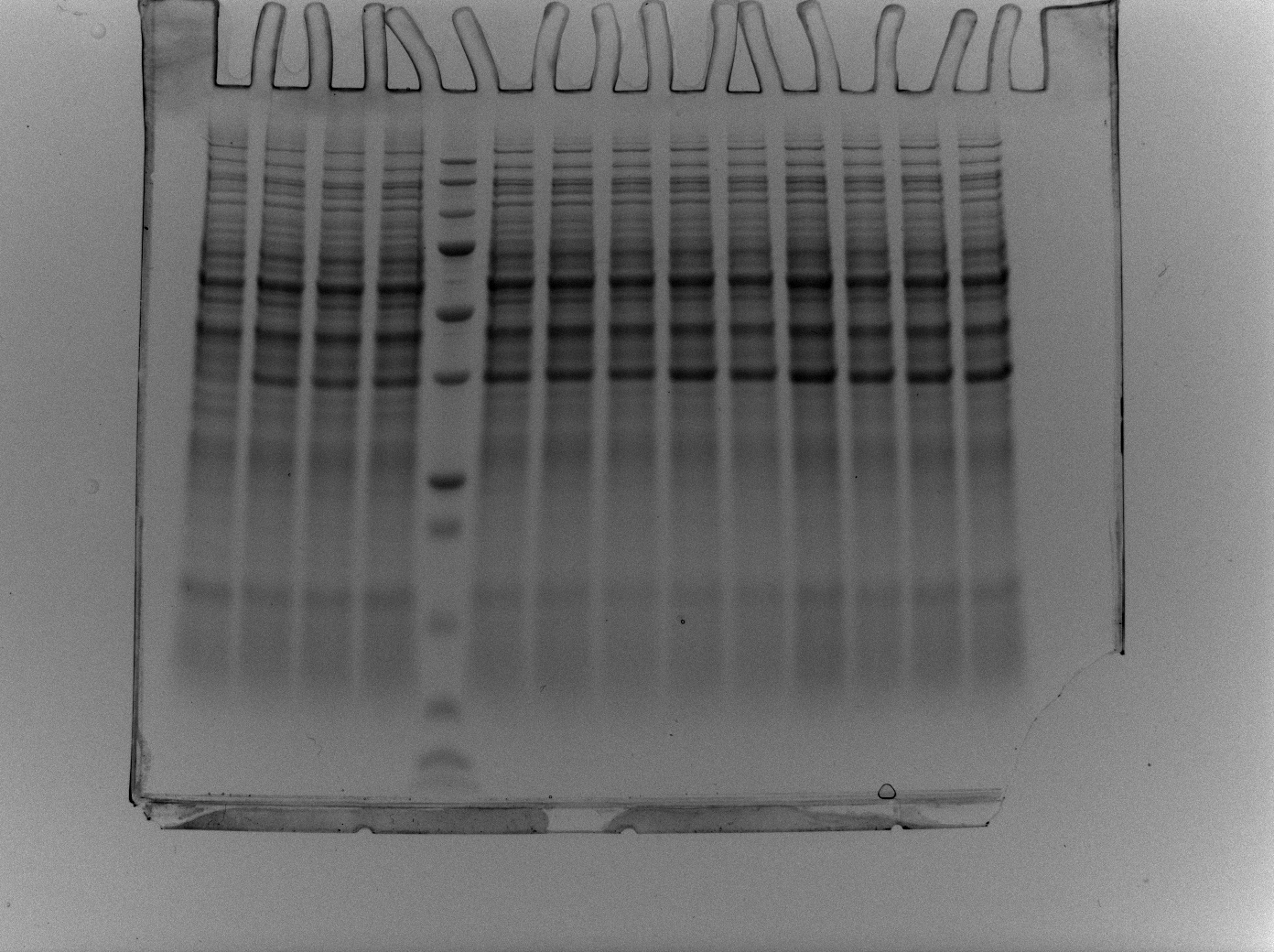

Supplement: Supplementary file 6 — Supplementary Data S5 [file 41598_2019_44500_MOESM6_ESM.zip › D2_SupDataS5/Fig4_raw_image/02_21222584_C.jpg]

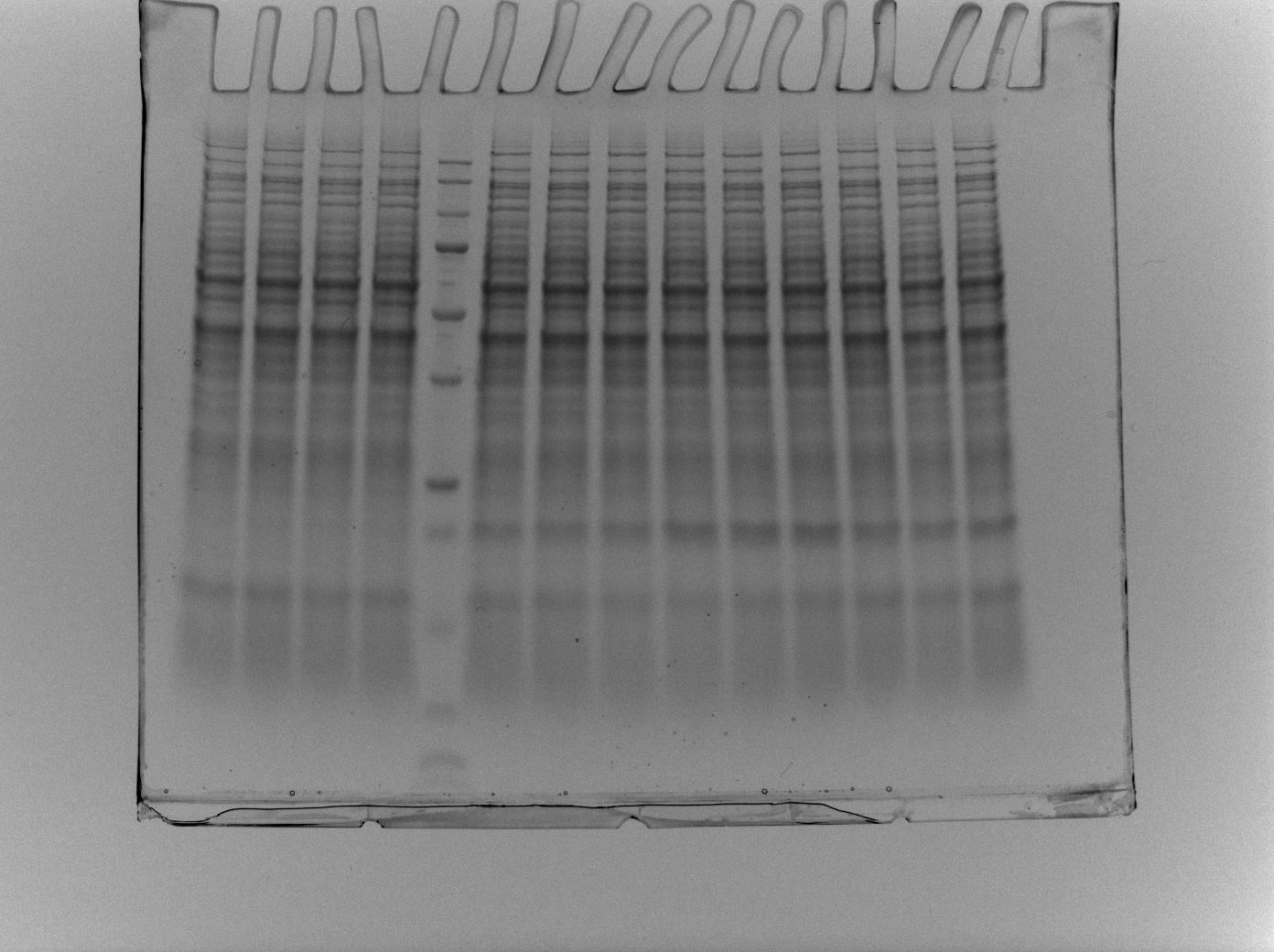

Supplement: Supplementary file 6 — Supplementary Data S5 [file 41598_2019_44500_MOESM6_ESM.zip › D2_SupDataS5/Fig4_raw_image/03_21224745_C.jpg]

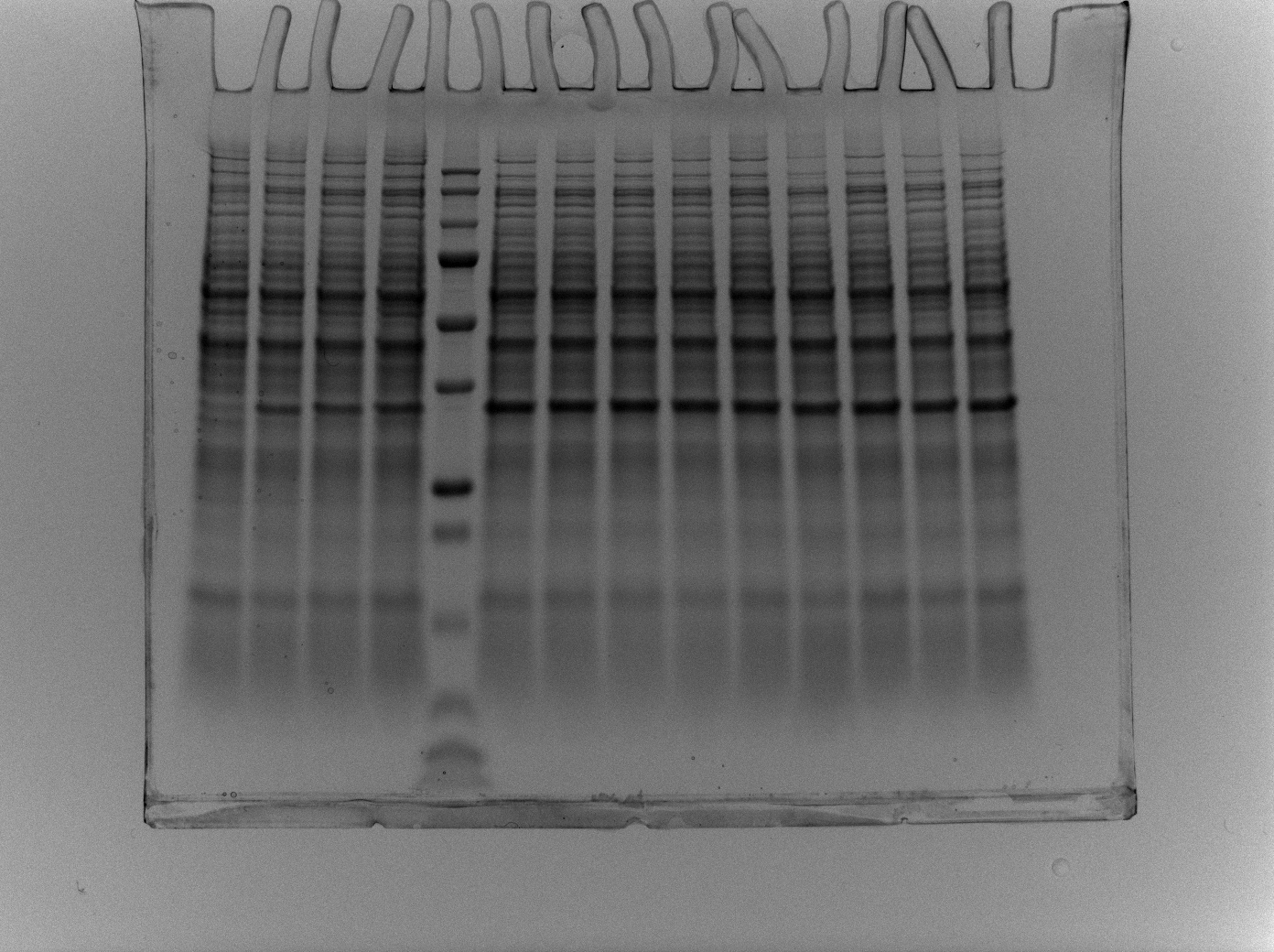

Supplement: Supplementary file 6 — Supplementary Data S5 [file 41598_2019_44500_MOESM6_ESM.zip › D2_SupDataS5/Fig4_raw_image/04_21220462_C.jpg]

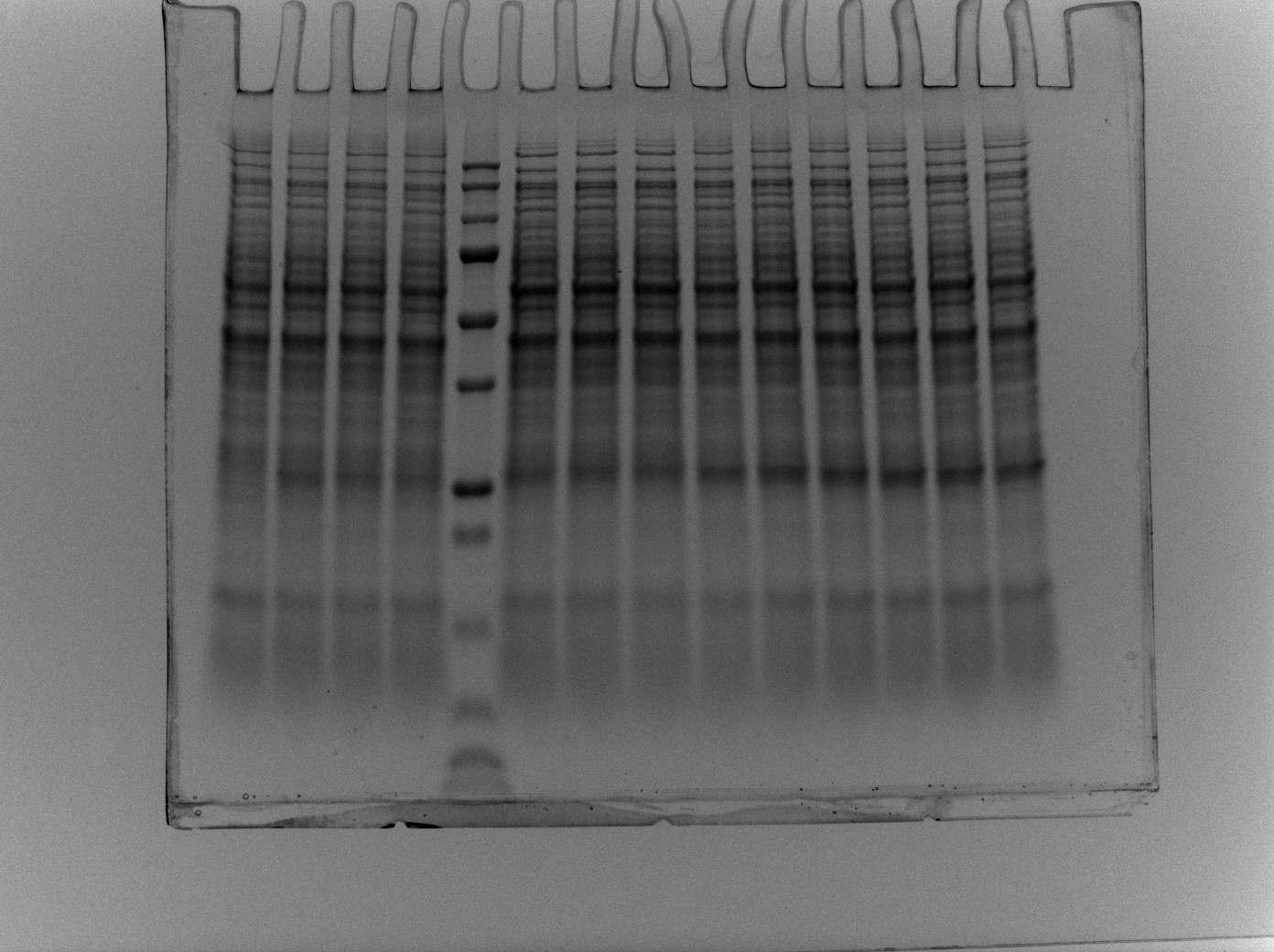

Supplement: Supplementary file 6 — Supplementary Data S5 [file 41598_2019_44500_MOESM6_ESM.zip › D2_SupDataS5/Fig4_raw_image/05_21220656_C.jpg]

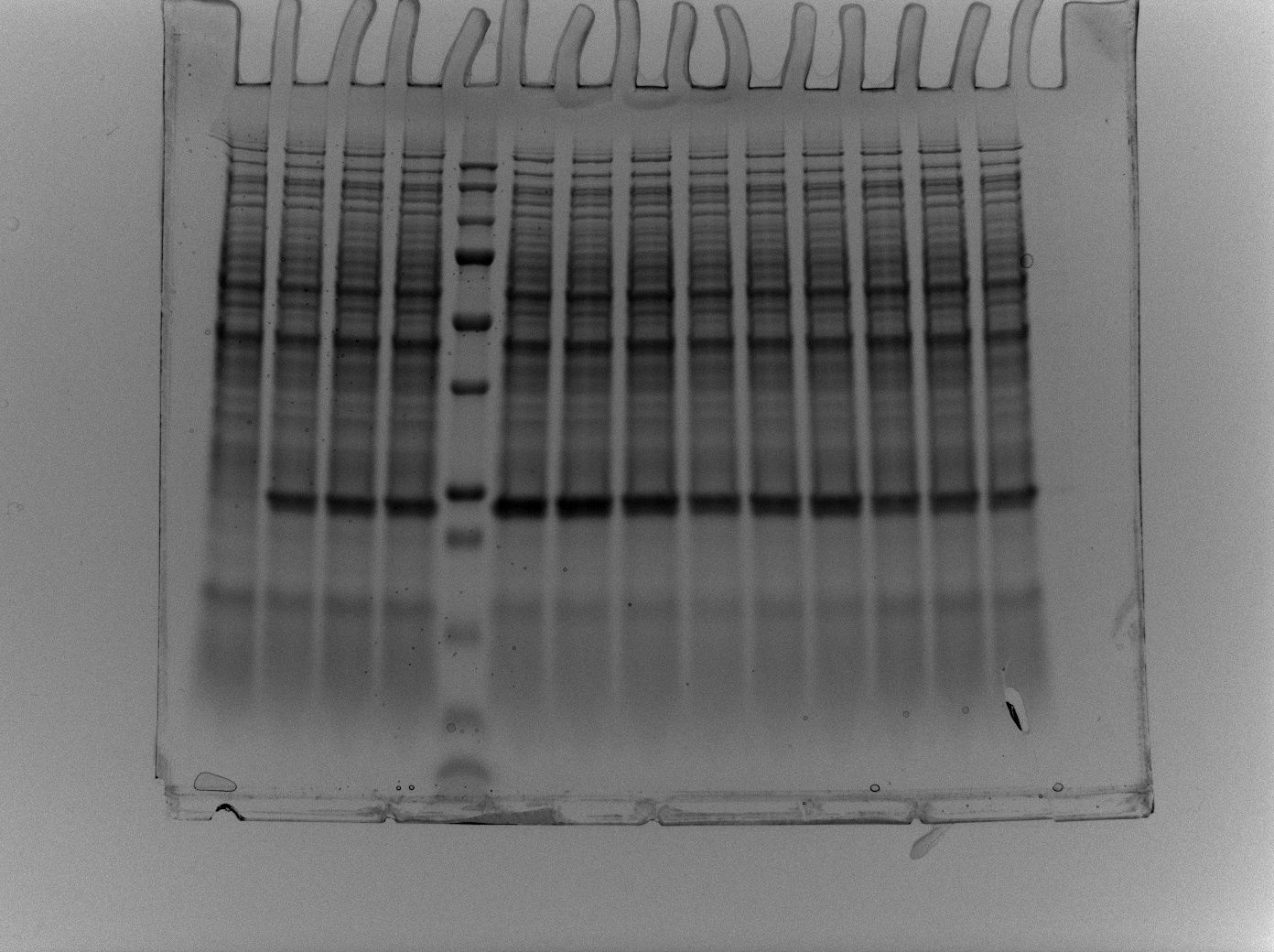

Supplement: Supplementary file 6 — Supplementary Data S5 [file 41598_2019_44500_MOESM6_ESM.zip › D2_SupDataS5/Fig4_raw_image/06_21222242_C.jpg]

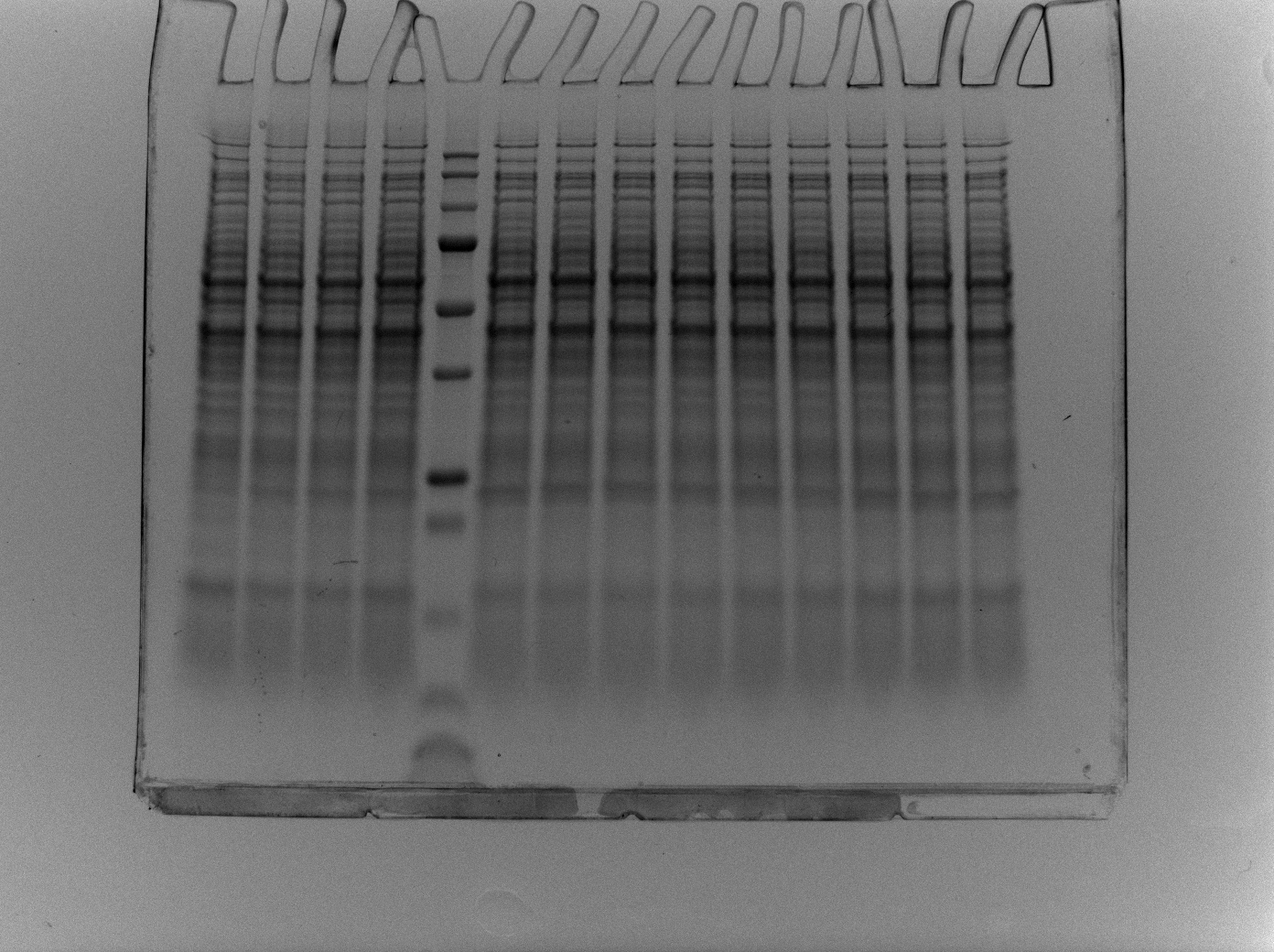

Supplement: Supplementary file 6 — Supplementary Data S5 [file 41598_2019_44500_MOESM6_ESM.zip › D2_SupDataS5/Fig4_raw_image/07_21226036_C.jpg]

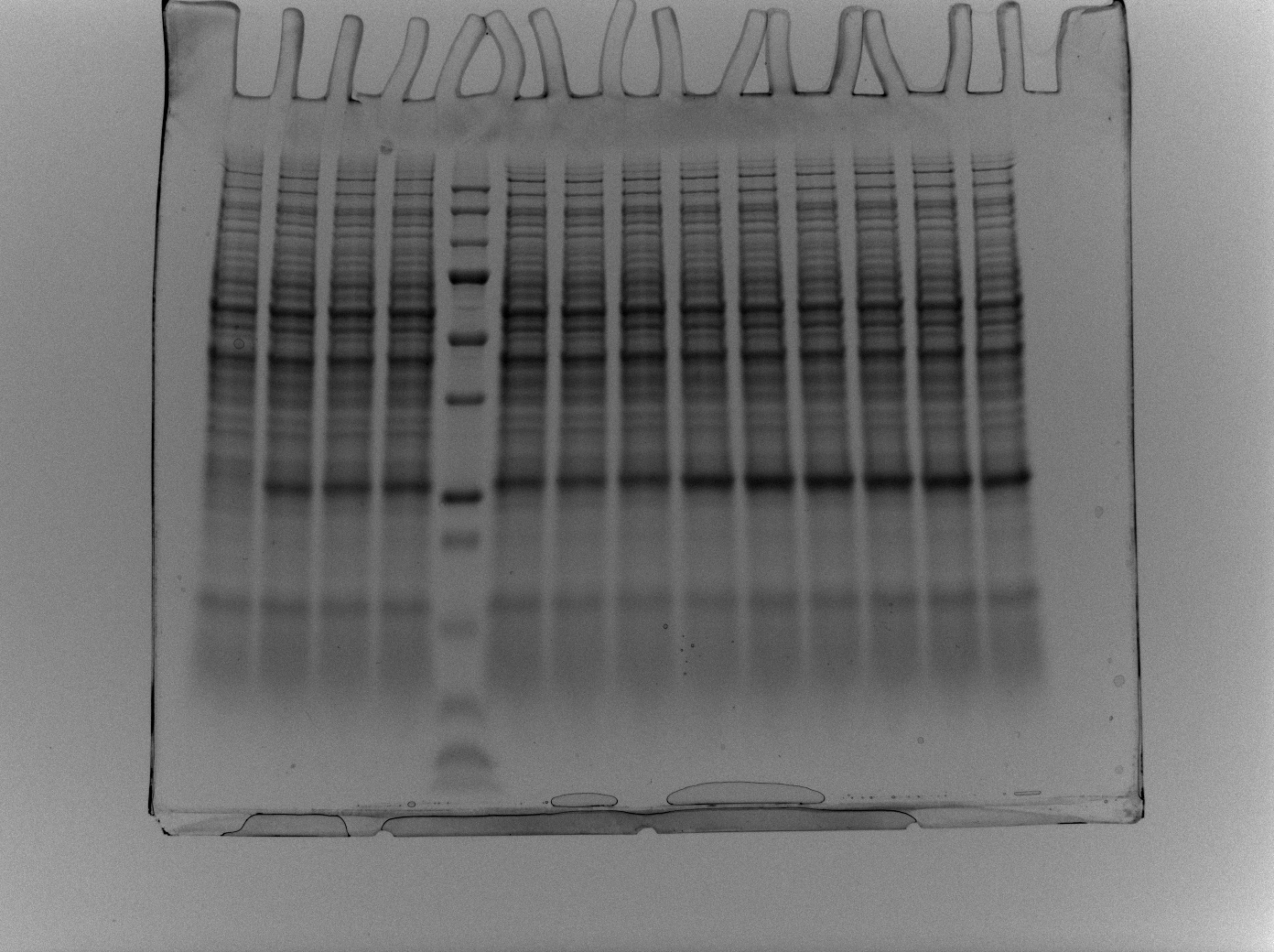

Supplement: Supplementary file 6 — Supplementary Data S5 [file 41598_2019_44500_MOESM6_ESM.zip › D2_SupDataS5/Fig4_raw_image/08_21220194_C.jpg]

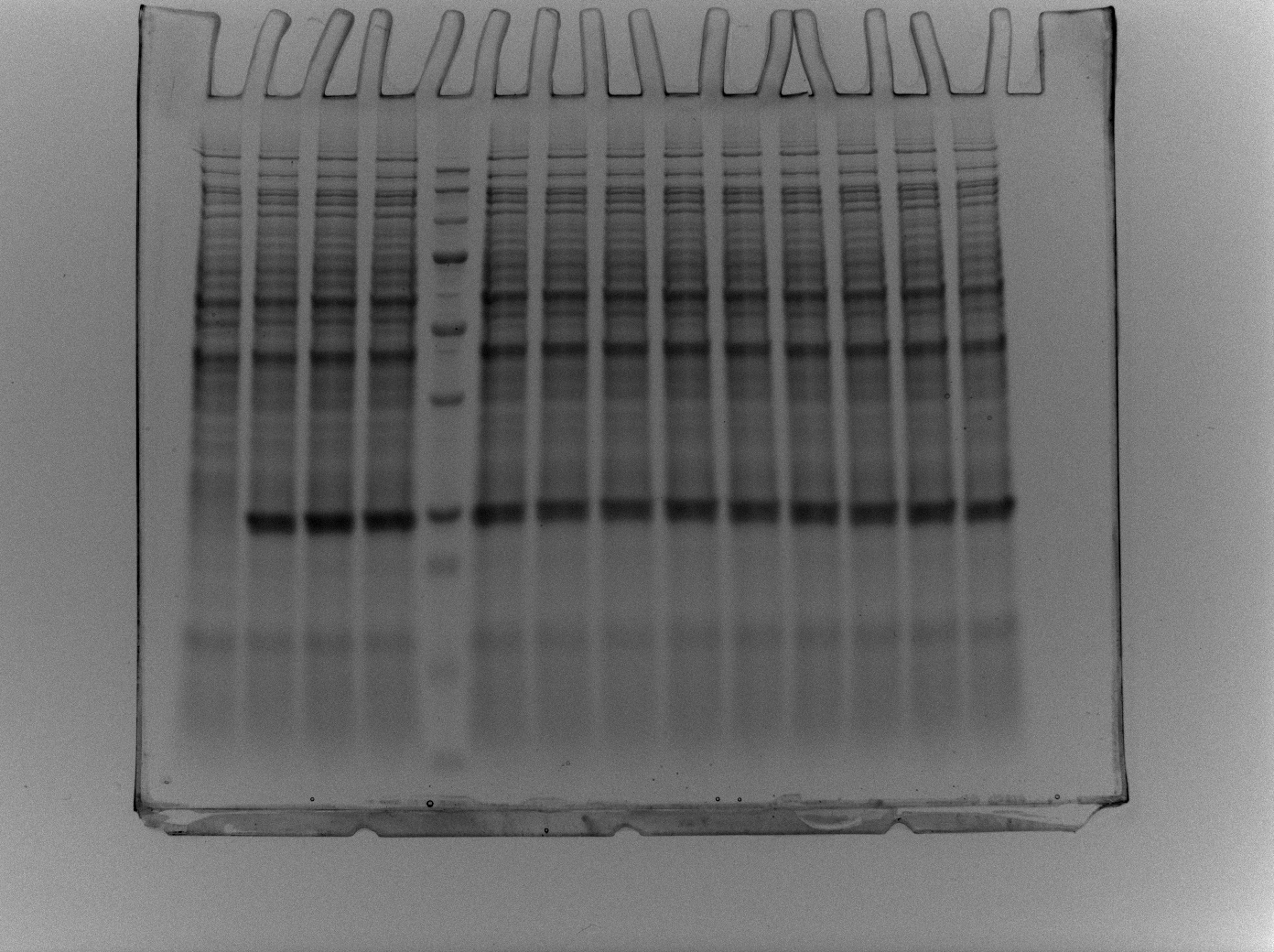

Supplement: Supplementary file 6 — Supplementary Data S5 [file 41598_2019_44500_MOESM6_ESM.zip › D2_SupDataS5/Fig4_raw_image/09_21220528_C.jpg]

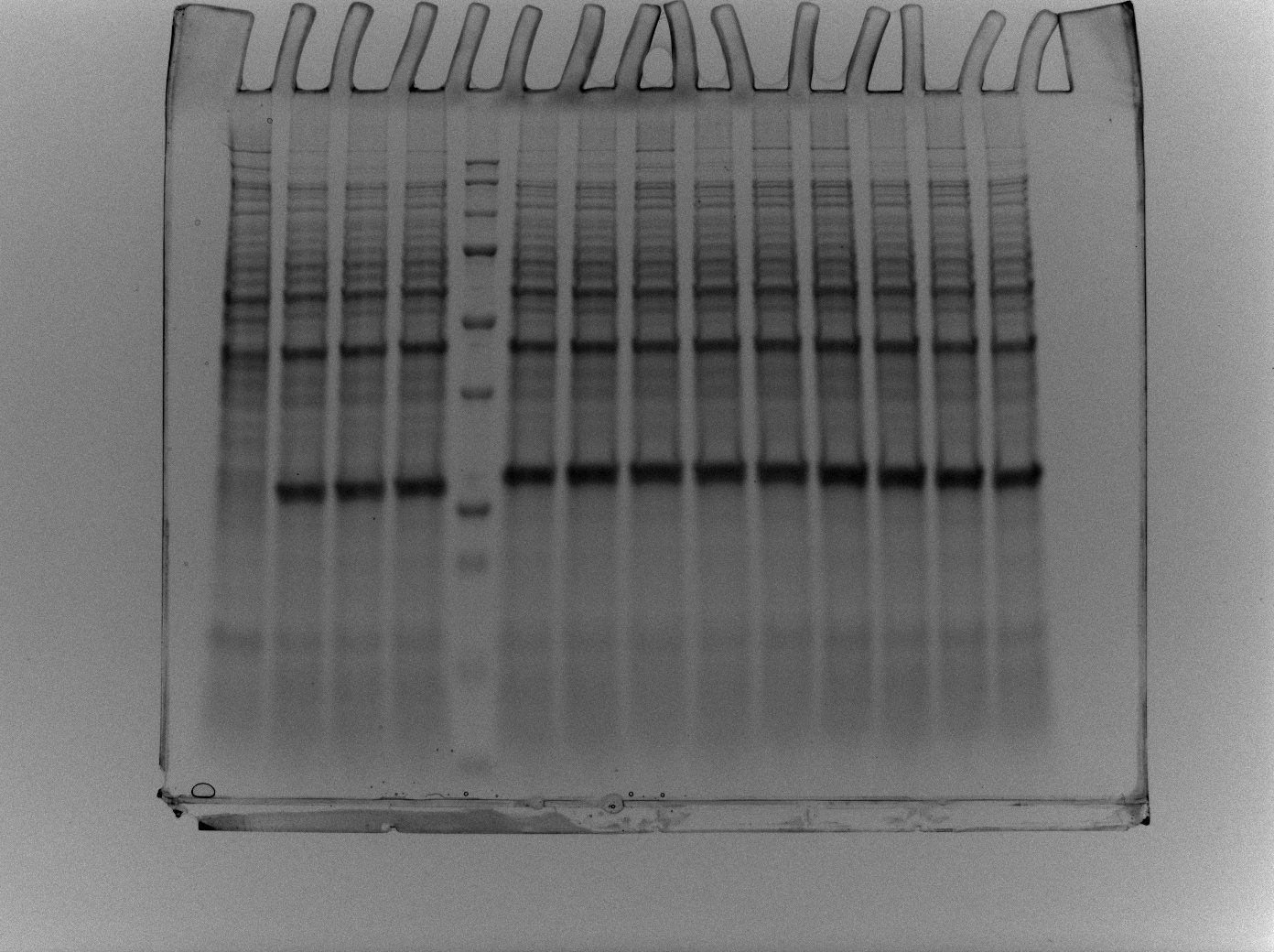

Supplement: Supplementary file 6 — Supplementary Data S5 [file 41598_2019_44500_MOESM6_ESM.zip › D2_SupDataS5/Fig4_raw_image/10_21222390_C.jpg]

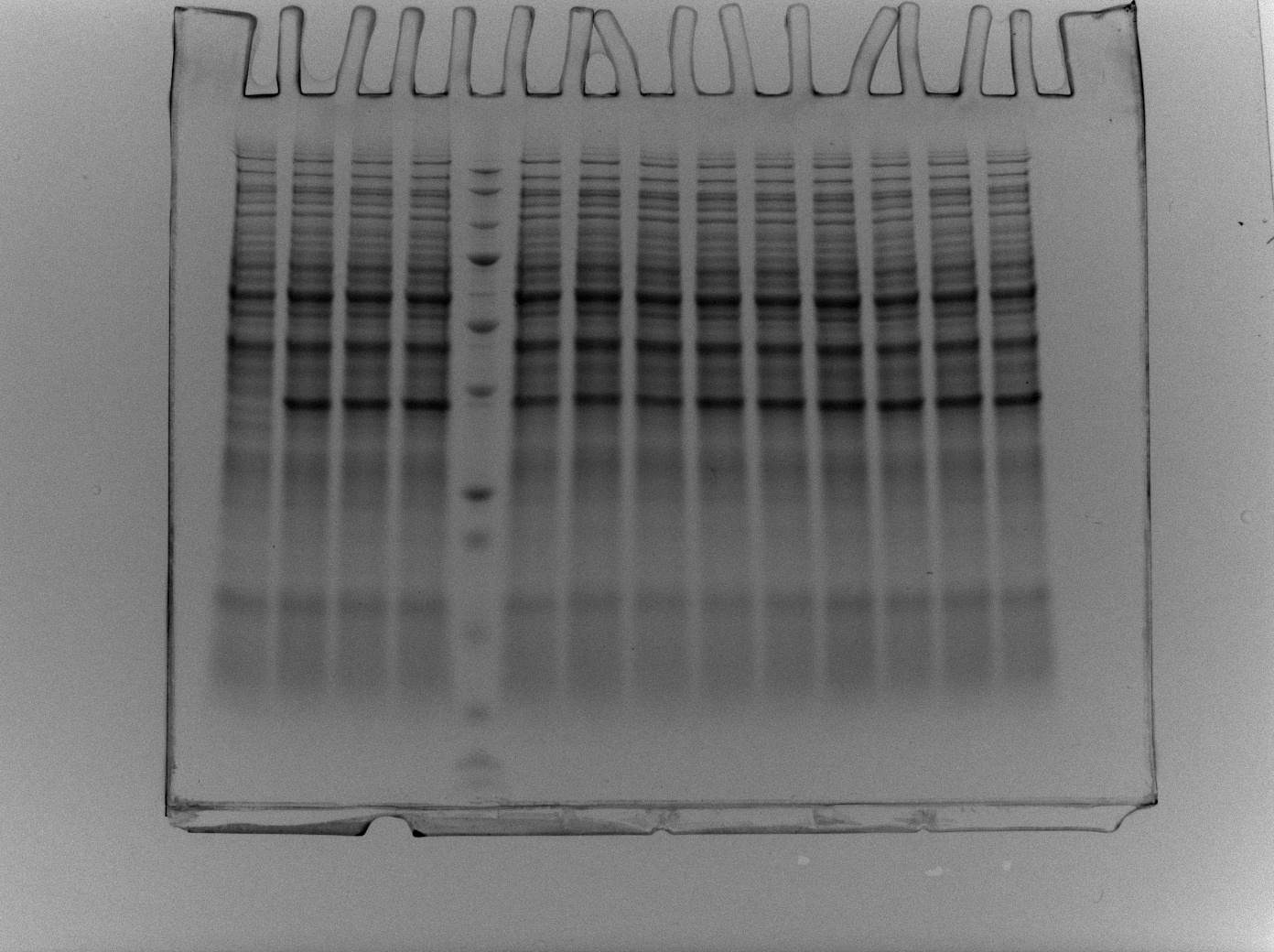

Supplement: Supplementary file 6 — Supplementary Data S5 [file 41598_2019_44500_MOESM6_ESM.zip › D2_SupDataS5/Fig4_raw_image/11_21224627_C.jpg]

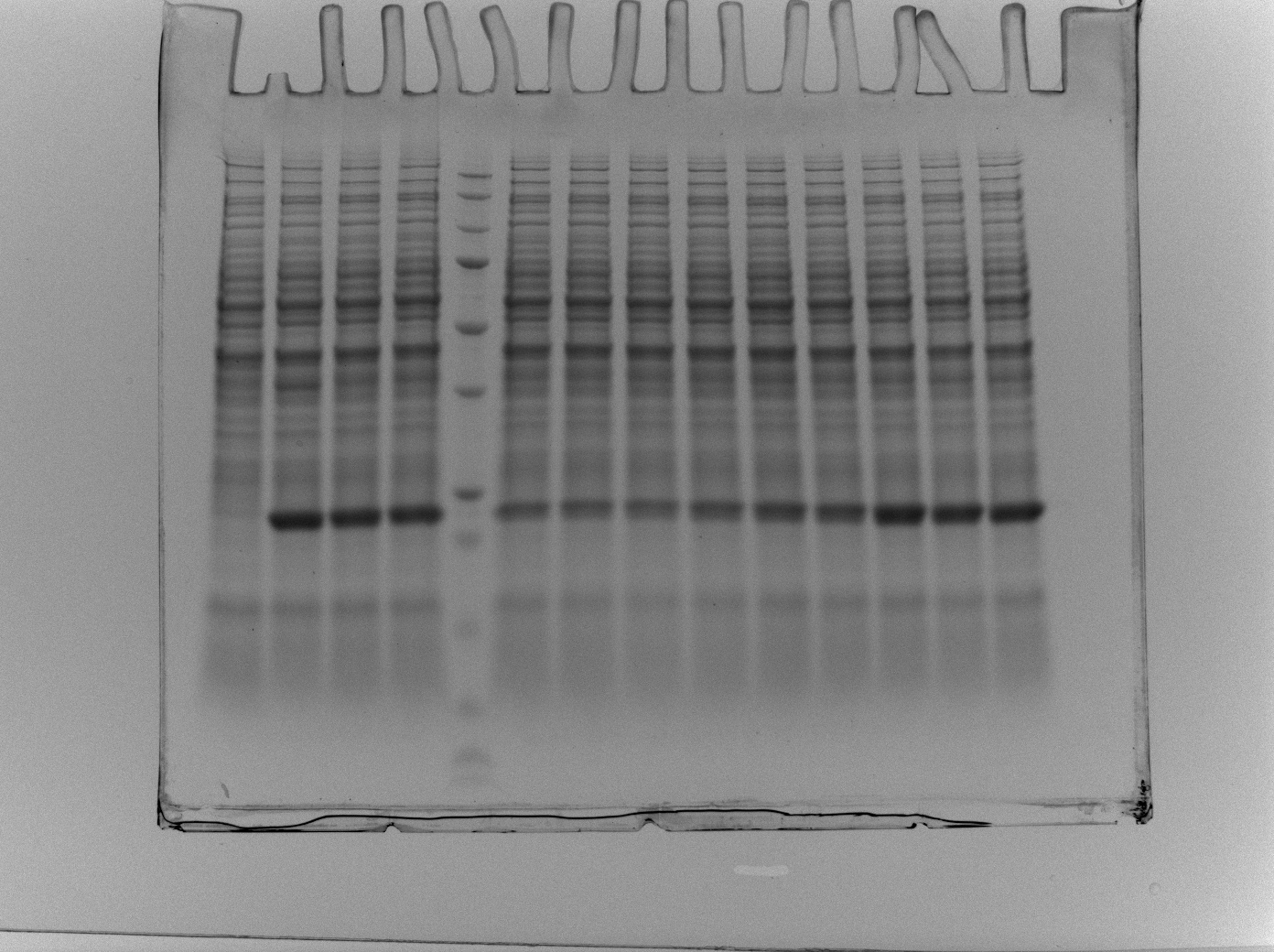

Supplement: Supplementary file 6 — Supplementary Data S5 [file 41598_2019_44500_MOESM6_ESM.zip › D2_SupDataS5/Fig4_raw_image/12_21224245_C.jpg]
